# Supplementary material for: Bayesian data assimilation for estimating instantaneous reproduction numbers during epidemics: Applications to COVID-19
Source: PLoS Comput Biol. 2022 Feb 23;18(2):e1009807. doi: 10.1371/journal.pcbi.1009807 (PMC8923496; doi:10.1371/journal.pcbi.1009807)
Supplement: S1 Text — Fig A. Illustrations of three types of observations and corresponding distributions of delay from the real infection date and observation. Fig B. Comparison between the simulation results using Poisson likelihood and Gaussian likelihood in DARt (both with 95% CrI). Fig C. Comparison between the estimated daily infection. The estimated infection by DARt is drawn in black with 95% CrI. The ground-truth simulated infection is in red and the back calculated infection is in yellow. Fig D. Comparison of estimated Rt curves of Hong Kong using different observations. Subplot A) shows Rt estimations (in black) from confirmed cases (in yellow). Subplot B) shows Rt estimations (in black) from daily onset (in yellow). Fig E. Epidemic dynamics in London, Leicester, Birmingham, Liverpool, Manchester, Sheffield, and Leeds. The top row of each subplot shows the number of daily observations (in yellow), the estimated daily observations (in blue) and the estimated daily infections (in green). The middle row shows the DARt results of Rt curve with 95% CrI (in black), while the probability of having abrupt changes is shown in the bottom row (i.e., Mt = 1) (in green). Fig F. The Rt estimation results under different levels of observation noise: A) N = 0, B) N = 1, C) N = 2 and D) N = 3, where the added Gaussian noise has the standard deviation equal to N times of the unperturbed observation. Fig G. The Rt estimation results of DARt with different truncation threshold: A) 0.01, B) 0.05 and C) 0.1. Fig H. A) The Rt estimation results of DARt obtained from the generation time and observation delay distributions with uncertainties. B) The Rt estimation results of DARt obtained from the generation time distribution following a Lognormal distribution. Table A. Transition probabilities of Mt*. Table B. Simulation results using synthetic data in the main manuscript. ΔRt-mean/ΔJt-mean and ΔRt-sd/ΔJt-sd are the mean and standard deviation of the differences between synthetic Rt/Jt and estimated R [file pcbi.1009807.s001.pdf]

Supplementary Materials

Bayesian data assimilation for estimating instantaneous reproduction numbers during epidemics: applications to COVID-19

Xian Yang, Shuo Wang, Yuting Xing, Ling Li, Richard Yi Da Xu,

Karl J. Friston and Yike Guo

Table of Contents

1 MATHEMATICAL MODELS 2

1.1 TIME-VARYING RENEWAL PROCESS 2
1.2 OBSERVATIONS OF THE TRANSMISSION DYNAMICS 5

2 MODEL INFERENCE 7

2.1 PROBLEM FORMULATION 7
2.2 INFERENCE AIMS 8
2.3 BAYESIAN UPDATING SCHEME 8
2.4 PARTICLE METHODS 13

3 DART APPLICATION TO HK 14

4 DART APPLICATION TO UK CITIES 15

5 EXPERIMENTAL SETTING 17

6 SENSITIVITY ANALYSIS 17

6.1 DIFFERENT LEVELS OF OBSERVATION NOISE 17
6.2 DIFFERENT VALUES OF TRUNCATION THRESHOLD 18
6.3 UNCERTAINTY IN THE DELAY DISTRIBUTIONS 19

7 SUPPLEMENTARY TABLE FOR SIMULATION RESULTS 21

REFERENCES 21

1 Mathematical Models

1.1 Time-varying Renewal Process

Origin of Instantaneous Reproduction Number $\boldsymbol{R}_t$. Both compartment models and time-since-infection models originate from the work of Kermack and McKendrick [1] and can be unified in the same mathematical framework [2]. Let us denote the numbers of susceptible and recovered individuals at calendar time t by $S(t)$ and $U(t)$ (recovered individuals are not denoted as $R(t)$ in order to avoid confusion with reproduction number). Taking into account of different phases of the infection period, we denote the number of infected individuals with an infection-age τ by $i(t,\tau)$. Thus, the overall number of currently infected individuals at time t is $I(t) = \int_0^t i(t,\tau)\, d\tau$ and the incident infection at time t is $j(t) = i(t,0)$. Governing equations of the homogenous transmission [2] are as follows:

$$\frac{dS(t)}{dt} = -\lambda(t)S(t) \tag{S1}$$

$$\left(\frac{\partial}{\partial t} + \frac{\partial}{\partial \tau}\right) i(t,\tau) = -\gamma(\tau)i(t,\tau) \tag{S2}$$

$$\frac{dU(t)}{dt} = \int_0^t \gamma(\tau)i(t,\tau)\, d\tau \tag{S3}$$

$$i(t,0) = \lambda(t)S(t) \tag{S4}$$

where $\lambda(t)$ is the rate at which susceptible individuals get infected at time t. This is given by the infection rates per single infected individual $\beta(\tau)$ with an infection-age τ and the number of infected individuals $i(t,\tau)$ as:

$$\lambda(t) = \int_0^t \beta(\tau)\, i(t,\tau)\, d\tau \tag{S5}$$

Similarly, $\gamma(\tau)$ is defined as the recovery rate with at infection-age τ. By simplifying Equation (S2) on the characteristic line ($t = \tau + c$), we have:

$$\frac{di(\tau + c, \tau)}{d\tau} = -\gamma(\tau) i(\tau + c, \tau) \tag{S6}$$

The solution to this ordinary differential equation is:

$$i(\tau + c, \tau) = i(c, 0)\mathcal{T}(\tau) \tag{S7}$$

where

$$\mathcal{T}(\tau) = exp\left(-\int_0^\tau \gamma(\sigma)\, d\sigma\right) \tag{S8}$$

Thus, we can link the infections with infection-age τ at time t to the incident infection at time $t - \tau$:

$$i(t, \tau) = \mathcal{T}(\tau)\, i(t - \tau, 0) \tag{S9}$$

By substituting Equation (S5) and (S9) into Equation (S4), the incident infection $j(t)$ at time t is

$$j(t) = \int_0^t S(t)\, \beta(\tau)\, \mathcal{T}(\tau)\, j(t - \tau) d\tau \tag{S10}$$

Then we have the infectiousness profile $\beta(t, \tau)$, representing the effectiveness rate at which an infectious individual with infection-age τ produces secondary cases at time t:

$$\beta(t, \tau) = S(t)\, \beta(\tau)\, \mathcal{T}(\tau) \tag{S11}$$

The corresponding instantaneous reproduction number $R(t)$ is derived from the integral of infectiousness profile $\beta(t, \tau)$:

$$R(t) = \int_0^\infty \beta(t, \tau) d\tau \tag{S12}$$

It is the average number of people that someone infected at time t is expected to infect, if conditions remain unchanged (i.e. susceptible population, infectiousness rate, recovery rate). From the above derivation, we observe that the infectiousness profile $\beta(t,\tau)$ and corresponding instantaneous reproduction number R_t are composed of three factors: $S(t)$, $\beta(\tau)$ and $\mathcal{T}(\tau)$. $S(t)$ represents the depletion of susceptible individuals: the decline of $S(t)$ will reduce the susceptible population size leading to possible herd immunity. $\beta(\tau)$ represents the infectiousness of individuals with infection-age τ. This is related to biological (e.g. viral shedding) and behavioural (e.g. contact rates) factors. $\mathcal{T}(\tau)$ represents the recovery rate of individuals with infection-age τ: faster recovery will result in shorter infectiousness period and smaller reproduction number. All three factors, $S(t)$, $\mathcal{T}(\tau)$ and $\beta(\tau)$ can be altered by the implementation of control measures along with time.

Decomposition of the Infectiousness Profile. R_t is determined by the evolution of the infectiousness profile $\beta(t,\tau)$ according to the Equation (S12). Further, the infectiousness profile $\beta(t,\tau)$ can be rewritten as:

$$\beta(t,\tau) = R(t)\,w(t,\tau) \qquad \text{(S13)}$$

where $w(t,\tau) = \beta(t,\tau)/\int \beta(t,\tau)d\tau$ is called the distribution of generation time, representing the probability distribution of infection events as a function of infection-age τ. That is, the distribution of time interval between the primary infection and subsequent secondary infection. In principle, the distribution of generation time is time-varying due to the three factors in Equation (S11), which increases the complexity of parametric modelling. Most existing studies assume a time-invariant generation time distribution (i.e. $w(t,\tau) = w(\tau)$) while the introduction of control measures results in the change of $R(t)$. Under this assumption, Equation (S10) can be rewritten as:

$$j(t) = R(t) \int_0^t w(\tau)\, j(t-\tau)\, d\tau \tag{S14}$$

This is the core formula for R_t estimation from the infection data. That is,

$$R(t) = j(t) / \int_0^t w(\tau)\, j(t-\tau)\, d\tau \tag{S15}$$

Or, we can have the corresponding discretised version:

$$R_t = \frac{j_t}{\sum_{k=1}^{T_w} w_k j_{t-k}} \tag{S16}$$

where T_w is the time span of the set $\{w_k\}$. This decomposition of infectiousness profile into R_t and time-invariant generation time distribution w_k is one of the fundamental formulae for R_t estimation in the existing literature (e.g. the well-known package 'EpiEstim' [3] and this paper).

1.2 Observations of the Transmission Dynamics

Formulation of the Observation Function. The infection number j_t is the ideal data source for R_t estimation according to Equation (S16). However, it is impossible to obtain the exact number of real-time infections through intensive screening. Instead, the infections are usually observed from the statistical reports of related events (i.e. epidemic curves) such as the daily report of confirmed cases, onset cases and death number. There is an inevitable delay between the occurrence of infecting events and the events being reported. That means these epidemic curves do not reflect the current incidence of infection j_t. We clarify this by formulating the observation function of the transmission dynamics. In the framework of data assimilation, j_t is the state variable of the dynamic epidemic system and its update is driven by the parameter R_t as described by Equation (S16). The aggregated reports C_t (e.g., daily confirmed cases, deaths) are the observing results of the state variable through an observation function H:

$$C_t = H(j_t) \tag{S17}$$

where H is the observation function and C_t is the observation result.

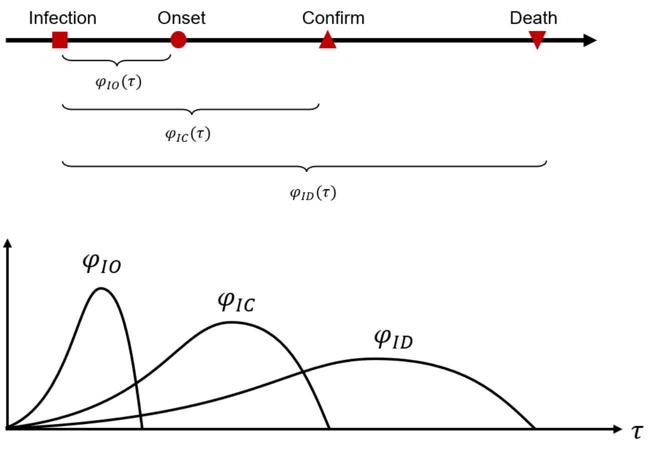

Fig A. Illustrations of three types of observations and corresponding distributions of delay from the real infection date and observation.

Observation Functions for Various Reports. The format of the observation function H depends on the type of reported data being used. In general, H is a convolutional operation summing up the portion of infected cases weighted by the distribution of time delay between being infected and being reported. Confirmed reports C_t^C, onset cases C_t^O and death reports C_t^D are the three most used reported data for R_t estimation [4–6] (illustrated in Fig A in S1 Text).

A. Onset Cases Reports. The reports of onset cases are usually compiled retrospectively from epidemic surveys of confirmed cases, which can be represented as:

$$C_t^O = \sum_{k=d_O}^{T_O} j_{t-k}\varphi_k^{IO} = \varphi^{IO} \otimes j_t \tag{S18}$$

where φ_k^{IO} is the probability that the symptom onset occurs k days after the initial infection date for a reported case, and d_O indicates the C_t^O can only cover information of infections at least d_O days before t. The value of d_O is determined by φ^{IO}. The distribution φ^{IO} is determined by the biological factors of the virus and has been investigated in the previous reports [7], which is considered time-independent. We use the symbol $\otimes$ to denote the convolution operation.

B. Confirmed Cases Reports. The epidemic curve of daily confirmed cases C_t^C is observed from

$$C_t^C = \sum_{k=d_C}^{T_C} j_{t-k}\varphi_k^{IC} = \varphi^{IC} \otimes j_t \tag{S19}$$

where φ_k^{IC} is the probability that a confirmed case is reported k days after the initial infection date, and d_C has a similar definition with d_O. The distribution φ^{IC} includes two parts: the time between infection to symptom onset φ^{IO}, and the time between symptom onset to reported confirmation φ^{OC}:

$$\varphi^{IC} = \varphi^{IO} \otimes \varphi^{OC} \tag{S20}$$

The former part φ^{IO} is usually similar across regions while the latter time delay φ^{OC} varies a lot due to test policies and screening capabilities.

C. Death Reports. The epidemic curve of death C_t^D is observed from

$$C_t^D = \rho_D \sum_{k=d_D}^{T_D} j_{t-k}\varphi_k^{ID} = \rho_D \varphi^{ID} \otimes j_t \tag{S21}$$

where ρ_D is the observed mortality rate of infected cases, φ_k^{ID} is the probability that a confirmed case is reported dead k days after the initial infection date, and d_D has a similar definition with d_O. ρ_D and φ^{ID} vary across different countries and periods due to capacities of treatment [5].

2 Model Inference

2.1 Problem Formulation

The time-varying renewal process can be formulated through the framework of state space hidden Markov models. The instantaneous reproduction number R_t and daily incident infections j_t are the two latent variables of the state space models, whose dependence is described by Equation (S16). Consider two evolution modes of R_t: emerging smooth changes

when interventions are being steadily introduced/relaxed, and undergoing an abrupt change due to intensive interventions (e.g., lockdown). We introduce another latent variable M_t to automate the switch between these two modes, which will be discussed in detail in the next section. The observations C_t are the observed results of j_t through Equation (S18), (S19) and (S21). We are interested in inferring the evolution of R_t (along with j_t and M_t) upon the real-time update of observations C_t.

2.2 Inference Aims

As revealed in Equation (S14) and Equation (S17), the observations experience time delay with respect to the update of the latent state, due to the lagging and averaging effects of convolution in Equation (S18)-(S21). Thus, the changes of R_t cannot be reflected in time, due to the incubation time and observation delay. In other words, accurate estimation of R_t at time t relies on future observations, which imposes the challenges of timely estimation. Therefore, we focus on two inference aims:

1. Given the latest observation, how to give a near real-time estimate of R_t and – equally if not more important – how to assess the uncertainty of the results?
2. Upon update of the real-time observations, how to modify estimations at all previous time steps and assess the uncertainties to make them more accurate taking into account the new information?

These two aims correspond to the two fundamental problems in Bayesian updating, namely the **filtering** and **smoothing** problems to be discussed in the next section.

2.3 Bayesian Updating Scheme

$\boldsymbol{X}_t =< R_{t^*}, \boldsymbol{J}_{t^*}, M_{t^*} >$ is defined as the latent state observed by C_t at time t. Since there is a delay between observation and infection, we suppose the most recent infection that can be observed by C_t is at the time $t^* = t - d$, where d is a constant determined by the distribution of observation delay. Suppose T_φ is the length of the vector $\boldsymbol{J}_{t^*} = [j_{t^*-T_\varphi+1}, j_{t^*-T_\varphi+2}, \cdots, j_{t^*}]$

such that C_t is only relevant to $\boldsymbol{J}_{t^*}$ via Equation (S18)-(S21) and j_{t^*} only depends on $\boldsymbol{J}_{t^*-1}$ via the renewal process. We formulate the estimate of the latent state $\boldsymbol{X}_t$ from the observed reports C_t as a data assimilation problem. A sequential Bayesian approach is adopted to infer time-varying latent state, which are composed of two phases: forward filtering and backward smoothing.

Forward filtering: A sequential Bayesian updating approach is employed to infer the latest latent state from the real-time observations. Let us denote the observation history between time 1 and t as $C_{1:t} = [C_1, C_2, \dots, C_t]$. Given that previous estimation $p(\boldsymbol{X}_{t-1}|C_{1:t-1})$ and new observation C_t, we would like to update the estimation of $\boldsymbol{X}_t$, i.e., $p(\boldsymbol{X}_t|C_{1:t})$ following Bayes' rule:

$$p(\boldsymbol{X}_t|C_{1:t}) = \frac{p(C_t|\boldsymbol{X}_t)p(\boldsymbol{X}_t|C_{1:t-1})}{\int p(C_t|\boldsymbol{X}_t)p(\boldsymbol{X}_t|C_{1:t-1})\,d\boldsymbol{X}_t} \tag{S22}$$

where $p(\boldsymbol{X}_t|C_{1:t-1})$ is *prior* and $p(C_t|\boldsymbol{X}_t)$ is *likelihood*. The *prior* can be written in the marginalised format:

$$p(\boldsymbol{X}_t|C_{1:t-1}) = \int p(\boldsymbol{X}_t|\boldsymbol{X}_{t-1})p(\boldsymbol{X}_{t-1}|C_{1:t-1})\,d\boldsymbol{X}_{t-1} \tag{S23}$$

which utilised the Markovian properties. By substituting Equation (S23) to Equation (S22), we obtain the iterative update of $p(\boldsymbol{X}_t|C_{1:t})$ given the transition $p(\boldsymbol{X}_t|\boldsymbol{X}_{t-1})$ and *likelihood* $p(C_t|\boldsymbol{X}_t)$:

$$p(\boldsymbol{X}_t|C_{1:t}) = \frac{p(C_t|\boldsymbol{X}_t)\int p(\boldsymbol{X}_t|\boldsymbol{X}_{t-1})p(\boldsymbol{X}_{t-1}|C_{1:t-1})\,d\boldsymbol{X}_{t-1}}{\iint p(C_t|\boldsymbol{X}_t)\int p(\boldsymbol{X}_t|\boldsymbol{X}_{t-1})p(\boldsymbol{X}_{t-1}|C_{1:t-1})\,d\boldsymbol{X}_{t-1}d\boldsymbol{X}_t} \tag{S24}$$

The *likelihood* can be calculated assuming an observation is with Gaussian variance:

$$p(C_t|\boldsymbol{X}_t) \sim \mathcal{N}(H(\boldsymbol{X}_t), {\sigma_C}^2) \tag{S25}$$

where H is chosen accordingly to the types of reports and ${\sigma_C}^2$ is the variance of observation error that can be approximated empirically (detailed settings can be found in Supplementary Section 4). As the likelihood function has explicitly considered observation noise, the function

is more robust to noise compared to Poisson likelihood (used in EpiEstim). The results of using the Poisson likelihood for the same synthetic data as depicted in Fig 3 are shown in Fig B in S1 Text, showing the benefits of considering observation noise in the likelihood.

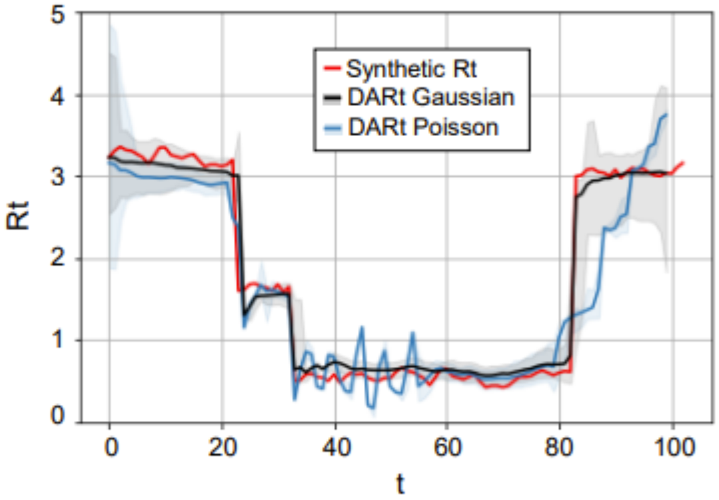

Fig B. Comparison between the simulation results using Poisson likelihood and Gaussian likelihood in DARt (both with 95% CrI).

Then we present the $p(\boldsymbol{X}_t|\boldsymbol{X}_{t-1})$, i.e., the transition of the latent state $\boldsymbol{X}_t = < R_{t^*}, \boldsymbol{J}_{t^*}, M_{t^*} >$ in details through analysing the evolution patterns of R_{t^*}. When R_{t^*} is evolving with smooth changes, we use a Gaussian random walk to model this pattern, named Mode I corresponding to $M_{t^*} = 0$. Under this mode, it is expected that R_{t^*} is similar to the previous time R_{t^*-1}. In contrast, the evolution of R_{t^*} can be altered significantly when intensive measures are induced. For example, R_{t^*} may experience an abrupt decrease due to the lockdown policy on time t^*. Under this circumstance, the epidemic history does not provide much information about the latest R_{t^*}, where we name it as Mode II corresponding to $M_{t^*} = 1$.

Formally, the evolution of R_{t^*} is described by the switching dynamics conditioned on M_{t^*}:

$$p(R_{t^*}|R_{t^*-1}) \sim \begin{cases} \mathcal{N}(R_{t^*-1}, {\sigma_R}^2) & M_{t^*} = 0 \quad \text{Mode I} \\ \mathrm{U}[0, R_{t^*-1} + \Lambda] & M_{t^*} = 1 \quad \text{Model II} \end{cases} \qquad \text{(S26)}$$

where $\mathcal{N}(R_{t^*-1}, {\sigma_R}^2)$ is a Gaussian distribution with the mean value of R_{t^*-1} and variance of ${\sigma_R}^2$, describing the random walk with the randomness controlled by σ_R. $\mathrm{U}[0, R_{t^*-1} + \Lambda]$ is a uniform distribution between 0 and $R_{t^*-1} + \Lambda$ allowing abrupt decrease while limiting the amount of increase. This is because we assume that R_{t^*} can undergo a big decrease when intervention is introduced but it is unlikely to have a dramatic increase in one day as the characteristics of disease would not change instantly.

In our model, we assume a discrete Markovian chain process for M_{t^*} with the transition probabilities listed in Table A in S1Text, meaning that the probability of having an abrupt change in R_{t^*} is low. This assumption is realistic as most of the time the R_{t^*} curve is undergoing smooth change.

Table A. Transition probabilities of M_{t^*}.

	$M_{t^*} = 0$	$M_{t^*} = 1$
$M_{t^*-1} = 0$	0.95	0.05
$M_{t^*-1} = 1$	0.95	0.05

Finally, we use the renewal process to provide transition of $\boldsymbol{J}_{t^*+1}$:

$$p(\boldsymbol{J}_{t^*}|\boldsymbol{J}_{t^*-1}, R_{t^*}) = Poisson(j_{t^*};\ R_{t^*} \sum_{k=1}^{T_w} w_k\, j_{t^*-k}\,) \prod_{m=1}^{T_\varphi - 1} \delta(\boldsymbol{J}_{t^*}^{(m)}, \boldsymbol{J}_{t^*-1}^{(m+1)}) \qquad \text{(S27)}$$

where $\boldsymbol{J}_{t^*}^{(m)}$ is the m-th component of the latent variable $\boldsymbol{J}_{t^*}$ and $\delta(x, y)$ is the Kronecker delta function. j_{t^*} is assumed to be drawn from a Poisson distribution with the mean equal to the prediction from the renewal process using R_{t^*} and $\boldsymbol{J}_{t^*-1}$. The overlaps between $\boldsymbol{J}_{t^*-1}$ and $\boldsymbol{J}_{t^*}$ are $\left\{\boldsymbol{J}_{t^*}^{(m)}\right\}_{m=1}^{T_\varphi-1} = \left\{\boldsymbol{J}_{t^*-1}^{(m+1)}\right\}_{m=1}^{T_\varphi-1} = [j_{t^*-T_\varphi+1}, \dots, j_{t^*-1}]$, whose distributions are assumed to be

consistent. By substituting Equation (S26) and (S27) and Table A in S1Text into Equation (S24), we have realised the sequential Bayesian update of the latent state $\boldsymbol{X}_t =< R_{t^*}, \boldsymbol{J}_{t^*}, M_{t^*} >$ for filtering.

Backward smoothing: To answer the second question on how to update previous estimations when more subsequent observations are available, we formulate it as a smoothing problem in the Bayesian updating framework. Based on the filtering results of $p(\boldsymbol{X}_t|C_{1:t})$, we can further achieve the smoothing results $p(\boldsymbol{X}_t|C_{1:T})$, where T is the time index of the last observation. To integrate the information from the subsequent observations, we use the backward pass method. First, the joint distribution $p(\boldsymbol{X}_1, \ldots, \boldsymbol{X}_T|C_{1:T})$ is decomposed as:

$$p(\boldsymbol{X}_1, \ldots, \boldsymbol{X}_T|C_{1:T}) = p(\boldsymbol{X}_T|C_{1:T}) \prod_{t=1}^{T} p(\boldsymbol{X}_t|\boldsymbol{X}_{t+1}, C_{1:T})$$

$$= p(\boldsymbol{X}_T|C_{1:T}) \prod_{t=1}^{T} p(\boldsymbol{X}_t|\boldsymbol{X}_{t+1}, C_{1:t}) \quad \text{(S28)}$$

where

$$p(\boldsymbol{X}_t|\boldsymbol{X}_{t+1}, C_{1:t}) = \frac{p(\boldsymbol{X}_{t+1}|\boldsymbol{X}_t)p(\boldsymbol{X}_t|C_{1:t})}{p(\boldsymbol{X}_{t+1}|C_{1:t})}. \quad \text{(S29)}$$

Then by integrating out $\boldsymbol{X}_1, \ldots \boldsymbol{X}_{t-1}, \boldsymbol{X}_{t+1}, \boldsymbol{X}_T$ in Equation (S28)

$$p(\boldsymbol{X}_t|C_{1:T}) = p(\boldsymbol{X}_t|C_{1:t}) \int p(\boldsymbol{X}_{t+1}|\boldsymbol{X}_t) \frac{p(\boldsymbol{X}_{t+1}|C_{1:T})}{p(\boldsymbol{X}_{t+1}|C_{1:t})} d\boldsymbol{X}_{t+1} \quad \text{(S30)}$$

which provides the iterative calculation of $p(\boldsymbol{X}_t|C_{1:T})$ from time T backwards to time t.

Our model includes both j_t and R_t into the latent state and jointly estimates them together. The Fig C in S1 Text compares j_t from our method with j_t by using the back calculation function (https://epiforecasts.io/EpiNow2/reference/backcalc_opts.html) from EpiNow2 (for the synthetic data mentioned in the main manuscript). We can see that our method returns a j_t curve with CrI that is closer to the simulated infection curve.

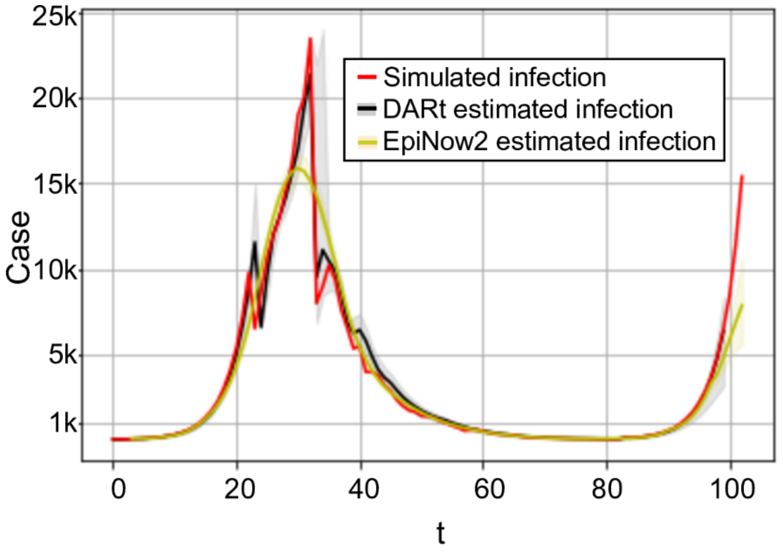

Fig C. Comparison between the estimated daily infection. The estimated infection by DARt is drawn in black with 95% CrI. The ground-truth simulated infection is in red and the back calculated infection is in yellow.

2.4 Particle Methods

The integrals in the filtering problem (Equation (S24)) and the smoothing problem (Equation (S30)) are intractable, thus we introduce a Sequential Monte Carlo (SMC) method called 'particle filter' to infer the latent state [8].

In Monte Carlo method, the continuous distribution of a random variable $X \sim \pi(x)$ is approximated by N independent samples with importance weights:

$$\pi(x) \approx \sum_{i=1}^{N} W^i \delta_{X^i}(x) \tag{S31}$$

where $\pi(x)$ is an arbitrary probability distribution and N independent samples $X^i \sim \pi(x)$ are drawn from the distribution with the normalized importance weight W^i. $\delta_{X^i}(x)$ denotes the Dirac delta mass located at the i-th sample X^i. These discrete samples are also called 'particles' in particle method, whose locations and weights are used to approximate the intractable integral. If x is a time-dependent state variable, we can update the samples to approximate the distribution through the Sequential Importance Sampling (SIS) technique [9]. The locations

and weights of the particles representing the target distribution are iteratively updated considering the new observations. For the filtering problem, we can set $p(\boldsymbol{X}_{1:t}|C_{1:t})$ as the target distribution and use N particles $\{\boldsymbol{X}_t^1, \boldsymbol{X}_t^2, \dots, \boldsymbol{X}_t^N\}$ with importance weight $\{W_t^1, W_t^2, \dots, W_t^N\}$ at time t. The SIS technique includes two steps: First, new positions of the particles $\boldsymbol{X}_t$ at time t are proposed according to a proposal function $q(\boldsymbol{X}_t|\boldsymbol{X}_{1:t-1})$ which can be the transition probability $p(\boldsymbol{X}_t|\boldsymbol{X}_{1:t-1})$. Next, the importance weight $\omega(\boldsymbol{X}_{1:t})$ of the proposed particles are calculated according to iteration in Equation (S22):

$$\omega(\boldsymbol{X}_{1:t}) = \frac{p(\boldsymbol{X}_{1:t}|C_{1:t})}{q(\boldsymbol{X}_{1:t})} = \frac{p(\boldsymbol{X}_{1:t-1}|C_{1:t-1})}{q(\boldsymbol{X}_{1:t-1})}\frac{p(\boldsymbol{X}_t|\boldsymbol{X}_{1:t-1})p(C_t|\boldsymbol{X}_t)}{q(\boldsymbol{X}_t|\boldsymbol{X}_{1:t-1})}$$

$$= \omega(\boldsymbol{X}_{1:t-1})\frac{p(\boldsymbol{X}_t|\boldsymbol{X}_{1:t-1})p(C_t|\boldsymbol{X}_t)}{q(\boldsymbol{X}_t|\boldsymbol{X}_{1:t-1})} = \omega(\boldsymbol{X}_{1:t-1})p(C_t|\boldsymbol{X}_t) \qquad \text{(S32)}$$

Therefore, the filtering results of $p(\boldsymbol{X}_{1:t}|C_{1:t})$ can be numerically approximated by the evolving particles and their importance weights. Similarly, the smoothing procedure of Equation (S30) can be approximated by the particles.

3 DARt Application to HK

Fig D in S1Text shows the results of R_t estimation using onsets and confirmed cases as observations in Hong Kong to estimate R_t, respectively. We choose Hong Kong for illustration purpose since both onset and reported confirmed cases are publicly accessible. The results from Fig D in S1Text reflect that, although the exact values of R_t at some time points would be different due to difference in observations, the overall trends are largely consistent. With a proper observation kernel according to the observation types (e.g., onset, confirmed cases), DARt can make generally consistent estimation from a wide range of observation types.

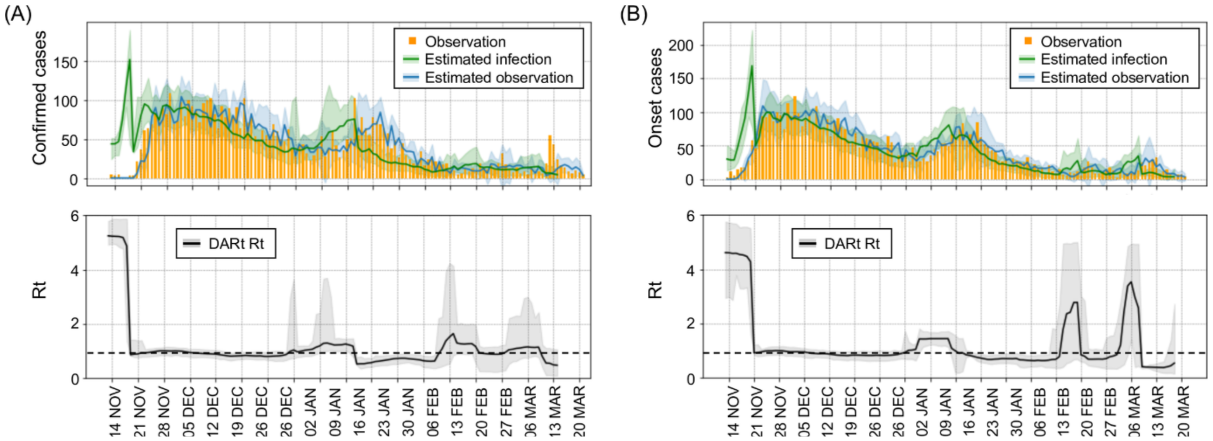

Fig D. Comparison of estimated R_t curves of Hong Kong using different observations. Subplot A) shows R_t estimations (in black) from confirmed cases (in yellow). Subplot B) shows R_t estimations (in black) from daily onset (in yellow).

4 DARt Application to UK Cities

We applied DARt to monitor seven cities in England as shown in Fig E in S1 Text, reflecting that the country-wide R_t curve shown in Fig 4C cannot be used to represent the epidemic dynamics across different local areas. With the application of DARt, we can examine the impacts of events, especially the easing of COVID restrictions that happened on May 17, 2021. Different cities responded to the easing of restrictions differently as reflected in their individual R_t curves given their different micro social structures. We can find that most cities have experienced an increase in R_t right after the easing of restrictions. In particular, sharp increases can be found in Leeds, Liverpool and Sheffield in late May. We can also find that the R_t levels for all cities were back to 1 in July and August. For London, which is one of the Euro Cup 2020 host cities, had an elevated R_t level during the Euro cup 2020. An obvious decrease in R_t, as indicated by M_t, happened around middle of June after the final of Euro Cup 2020. This observation implies big sport event would increase the R_t values.

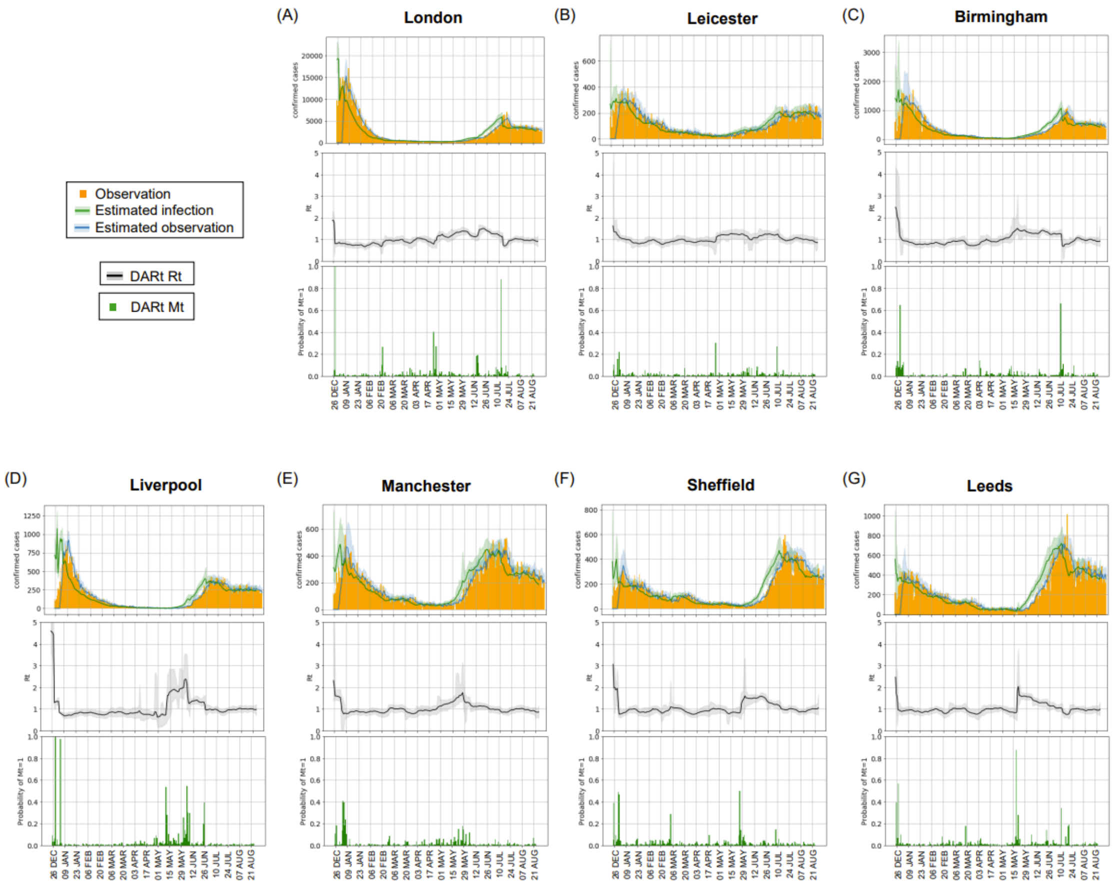

Fig E. Epidemic dynamics in London, Leicester, Birmingham, Liverpool, Manchester, Sheffield, and Leeds. The top row of each subplot shows the number of daily observations (in yellow), the estimated daily observations (in blue) and the estimated daily infections (in green). The middle row shows the DARt results of R_t curve with 95% CrI (in black), while the probability of having abrupt changes is shown in the bottom row (i.e., $M_t = 1$) (in green).

5 Experimental setting

In our experiments, the generation time and observation delay distributions are adopted from the previous reports [6,10]. To truncate these distributions into a fixed length, we discard the time points with the kernel values smaller than 0.1 resulting in the length of $\boldsymbol{J}_{\mathrm{t}}$ as 7. The initial guess of R_t at $t = 0$ is set to be uniformly distributed from 1 to 5. We set $\sigma = 0.1$ for getting smooth R_t change in Model I. In Model II, we set $\Lambda = 0.5$ for all regions. To implement the particle filter, the number of particles is set to 200 for approximating distributions.

The variance of observation error ${\sigma_C}^2$ is estimated empirically. We first calculate the 7-day moving average observations. By subtracting the moving average from the observation, we obtain a difference curve, approximating random observation fluctuations. The next step is to perform the 7-day moving average calculation again on the squared value of the difference curve, where the resulted curve is regarded as the observation error variance. Finally, we use a Gaussian distribution as the likelihood function (Equation (S25)), where its variance is approximated by the observation error variance curve.

For performance comparison, we choose two state-of-art methods which are EpiEstim and EpiNow2. For EpiNow2, we tried different modes including the default mode, the fast computation mode and the back-calculation based mode. All the modes show over-smoothed results. Thus, in the manuscript we show the results from the default settings.

6 Sensitivity Analysis

6.1 Different levels of observation noise

To investigate the robustness of different comparative methods, Fig F in S1 Text shows the R_t estimation results from DARt, EpiEstim and EpiNow2 under different levels of observation noise. We can find that with greater noises, all comparative methods would experience some

estimation errors. Particularly for EpiEstim, local fluctuations became obvious on the first few days. For DARt, although high observation noises would influence the M_t estimation results, the results are still informative to indicate abrupt changes.

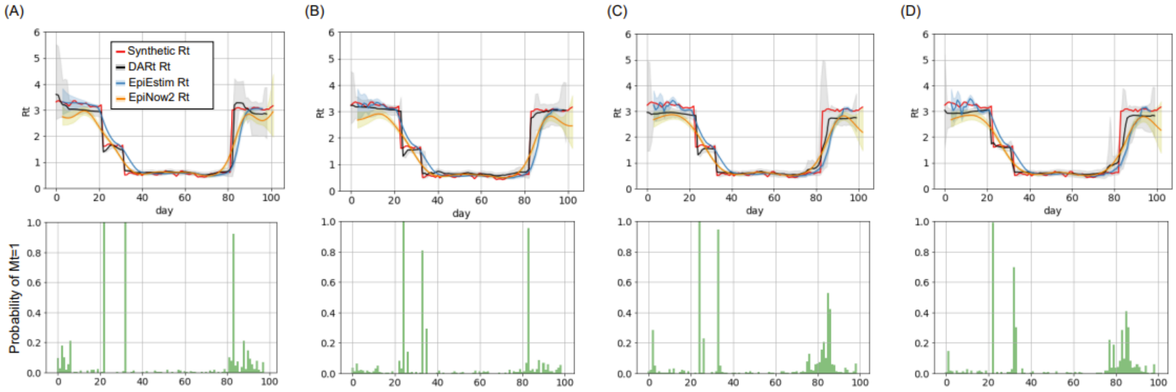

Fig F. The R_t estimation results under different levels of observation noise: A) N=0, B) N=1, C) N=2 and D) N=3, where the added Gaussian noise has the standard deviation equal to *N* times of the unperturbed observation.

6.2 Different values of truncation threshold

Here, we would like to conduct experiments to investigate how different settings of the truncation threshold would influence the R_t estimation results. The subplots (A), (B) and (C) of Fig G in S1 Text present the R_t results with the truncation threshold set to 0.1, 0.05 and 0.01, respectively. Other experimental settings are same as that in the main manuscript with the absence of observation noise. We can find that the results from these three subplots are quite similar implying that setting the threshold to be 0.1 as what we did throughout the paper would not influence the estimation significantly.

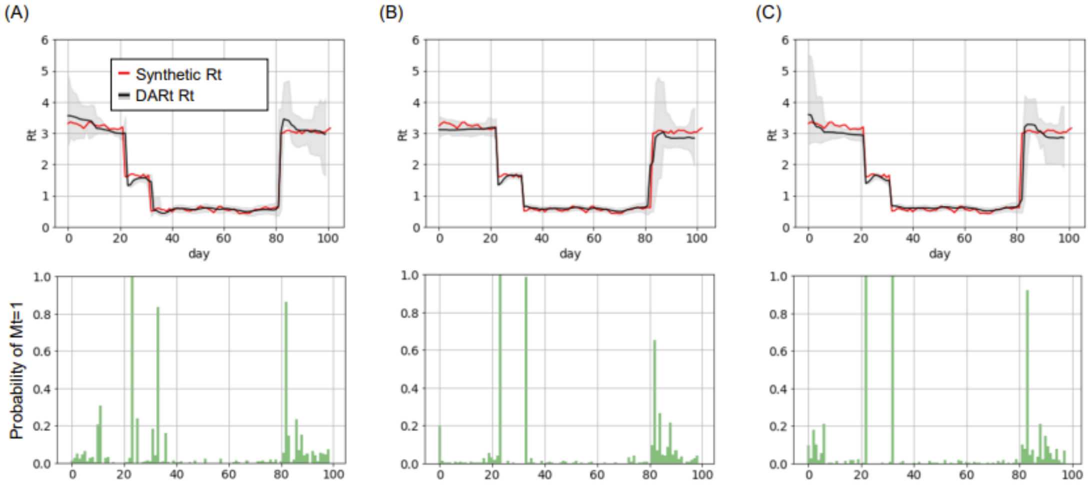

Fig G. The R_t estimation results of DARt with different truncation threshold: A) 0.01, B) 0.05 and C) 0.1.

6.3 Uncertainty in the delay distributions

In this paper, we focus on dealing with uncertainties brought by noisy observations. However, we are also quite interested in how our model would behave when uncertainties in the distributions of generation time and observation delay exist. Fig H (A) in S1 Text shows the R_t estimation results when the parameters of the generation time and observation delay distributions are set as follows: the distribution of generation time ~ Gamma(shape, scale), where $shape \sim \mathcal{N}(5.51, (10\% * 5.51)^2)$ and scale $\sim \mathcal{N}(0.81, (10\% * 0.81)^2)$; the distribution of observation delay distribution ~ Lognormal(mean, SD), where $mean \sim \mathcal{N}(1.64, (10\% * 1.64)^2)$ and $SD \sim \mathcal{N}(0.363, (10\% * 0.363)^2)$. We can see that the R_t estimation results from uncertain time distributions (The R function 'bootstrapped_dist_fit' used for fitting lognormal distribution is adopted from the EpiNow2 package) are still consistent with the synthetic R_t.

Fig H (B) in S1 Text further investigates the impact of choosing different forms of time distributions. Rather than assuming the generation time distribution follows a Gamma distribution, here we set it to be Lognormal. The parameters of the Lognormal distribution are obtained from fitting the original Gamma distribution by the Lognormal distribution[2]. The generation time distribution is hence set to be drawn from Lognormal(mean, SD), where $mean \sim \mathcal{N}(1.26, 0\ .042^2)$ and $SD \sim \mathcal{N}(0.46, 0.032^2)$. We can find that the estimated R_t results at the beginning and ending time points are lower than the synthetic R_t. This is because the generation time distribution used for estimation is different from the original distribution used in the synthetic data generation. However, the general trend of R_t with three abrupted changes is still well captured by DARt.

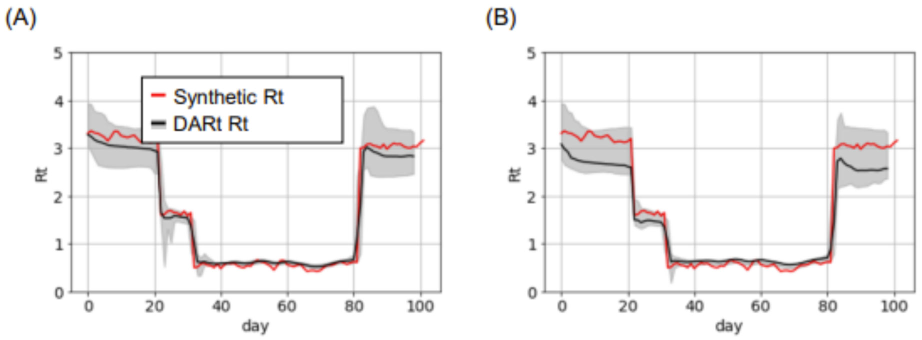

Fig H. A) The R_t estimation results of DARt obtained from the generation time and observation delay distributions with uncertainties. B) The R_t estimation results of DARt obtained from the generation time distribution following a Lognormal distribution.

7 Supplementary table for simulation results

The simulation results in Fig 3 from the main manuscript have illustrated the performance of DARt. As indicated in Table B in S1 Text, the simulation results further quantitively confirms our findings by calculating the mean and standard deviation of estimation differences over time. We can easily observe that: 1) compared with EpiNow2 and EpiEstim, DARt achieves much smaller estimation errors; 2) compared with DARt without smoothing, the full implementation of DARt with smoothing can reduce estimation errors, showing that smoothing has greatly contributed to uncertainty reduction.

Table B. Simulation results using synthetic data in the main manuscript. ΔR_t-mean/ΔJ_t-mean and ΔR_t-sd/ΔJ_t-sd are the mean and standard deviation of the differences between synthetic R_t/J_t and estimated R_t/J_t. Since EpiEstim does not estimate $\boldsymbol{J_t}$, we leave the corresponding values as NA.

	$\Delta \boldsymbol{R_t}$-mean	$\Delta \boldsymbol{R_t}$-sd	$\Delta \boldsymbol{J_t}$-mean	$\Delta \boldsymbol{J_t}$-sd
**EpiNow2**	0.30	0.30	588.98	1283.08
**EpiEstim**	0.21	0.39	NA	NA
**DARt without smoothing**	0.21	0.33	729.42	2301.83
**DARt**	0.12	0.14	333.83	681.07

References

1. Kermack WO, McKendrick AG. A contribution to the mathematical theory of epidemics. Proc R Soc Lond Ser Contain Pap Math Phys Character. 1927;115: 700–721. doi:10.1098/rspa.1927.0118

2. Chowell G, Hyman JM, Bettencourt LMA, Castillo-Chavez C. Mathematical and Statistical Estimation Approaches in Epidemiology. Chowell G, Hyman JM, Bettencourt LMA, Castillo-Chavez C, editors. Mathematical and Statistical Estimation Approaches in Epidemiology. Dordrecht: Springer Netherlands; 2009. doi:10.1007/978-90-481-2313-1

3. Cori A, Ferguson NM, Fraser C, Cauchemez S. A new framework and software to estimate time-varying reproduction numbers during epidemics. Am J Epidemiol. 2013;178: 1505–1512. doi:10.1093/aje/kwt133

4. Pan A, Liu L, Wang C, Guo H, Hao X, Wang Q, et al. Association of Public Health Interventions With the Epidemiology of the COVID-19 Outbreak in Wuhan, China. JAMA. 2020;323: 1915. doi:10.1001/jama.2020.6130

5. Flaxman S, Mishra S, Gandy A, Unwin HJT, Mellan TA, Coupland H, et al. Estimating the effects of non-pharmaceutical interventions on COVID-19 in Europe. Nature. 2020; 1–5.

6. Leung K, Wu JT, Liu D, Leung GM. First-wave COVID-19 transmissibility and severity in China outside Hubei after control measures, and second-wave scenario planning: a modelling impact assessment. The Lancet. 2020;395: 1382–1393. doi:10.1016/S0140-6736(20)30746-7

7. He X, Lau EHY, Wu P, Deng X, Wang J, Hao X, et al. Temporal dynamics in viral shedding and transmissibility of COVID-19. Nat Med. 2020. doi:10.1038/s41591-020-0869-5

8. Doucet A, Johansen AM. A tutorial on particle filtering and smoothing: Fifteen years later. Handbook of nonlinear filtering. 2009. p. 3.

9. Maceachern SN, Clyde M, Liu JS. Sequential importance sampling for nonparametric Bayes models: The next generation. Can J Stat. 1999. doi:10.2307/3315637

10. Ferretti L, Wymant C, Kendall M, Zhao L, Nurtay A, Abeler-Dörner L, et al. Quantifying SARS-CoV-2 transmission suggests epidemic control with digital contact tracing. Science. 2020;368: eabb6936. doi:10.1126/science.abb6936
