# Supplementary material for: Bayesian data assimilation for estimating instantaneous reproduction numbers during epidemics: Applications to COVID-19
Source: PLoS Comput Biol. 2022 Feb 23;18(2):e1009807. doi: 10.1371/journal.pcbi.1009807 (PMC8923496; doi:10.1371/journal.pcbi.1009807)
Supplement: Attachment — Submitted filename: Yang_et_al_review.pdf [file pcbi.1009807.s002.pdf]

In the manuscript entitled "Bayesian data assimilation for estimating transmission dynamics in computational epidemiology", the authors present an analytical framework that aims at addressing several common issues that epidemiologists face in the context of an epidemic outbreak. During such times, several metrics are used to characterize the spread of a pathogen. R_t, the "time-varying reproduction number", is one of them and can be defined as the average number of secondary cases spanning from an index case occurring at time *t*. The variations of Rt reflect directly on how aggressive the epidemic is at a given time step, and this measure is often used by policymakers to implement or alleviate control measures, as well as to assess their impact.

The manuscript provides a powerful new tool to the existing literature on estimating parameters of interest to assess epidemic outbreaks, with the added benefit of a public GitHub repository providing reproducible examples. I am overall convinced that this work could be published in PLoS Computational Biology, but major changes are necessary before acceptance.

As an outbreak unfolds, more and more data become available both with respect to the pathogen biology / life cycle / disease natural history, as well epidemic spread. These two elements pave the way towards building an epidemic model.

As a highlight, the authoes identify three main sources of biases in currently available methods:

1) Lagging: incident cases occurring at time *t* may not be observed immediately but after a random delay
2) Averaging inference: the estimated parameters are assumed constant within estimation windows
3) Uncertainty

The main addition of this work to the current epidemiologist toolbox is the methodological development around the forward estimation and backward smoothing of prior estimates as data accumulates. As a first proof of concept, the authors simulated controlled epidemic processes governed by calibrated values of Rt and compared the performance of their DARt method with that from EpiEstim (Cori *et al.*). After this simulated validation, they set out to assess the performance of DARt with COVID–19 incidence data from 4 regions/countries: Wuhan, Hong Kong, the United Kingdom and Sweden.

The writing should be heavily improved to *i)* ease readability and *ii)* clarify the scope of the work. Furthermore, while it provides undeniable methodological development, the statements about overall reduced uncertainty should be toned done to better reflect that uncertainty is better split between its main sources, and subsequently propagated into posterior distributions. In particular, the overall process of estimating reproduction numbers still makes use of the commonly reported generation time distribution approximated by serial interval; parametric or empirically-calibrated reporting delay as well as incubation time; underlying S(E)IR structure…

Last but not least, I am somewhat disturbed by some phrasing used to benchmark the performances of DARt against EpiEstim in the controlled simulations: the (overly-long) legend

for Figure 3 reads more as a negative critic towards EpiEstim where it should only emphasize that methodological developments helped at better capturing abrupt changes in epidemic process. This comment applies all throughout the text and I strongly suggest that previous works, conducted by different teams, be presented in a more respectful way.

Major comments

1) Inconsistency between manuscript title and overall content

The title misleads the reader into expecting the description of a general Bayesian framework for computational epidemiology. Instead, the manuscript focuses entirely on the COVID–19 outbreak that has kept every epidemiologist busy over the past ~1.5 year. While I obviously agree that in the present global sanitary context, COVID–19 is the leading cause of epidemiological model development and an excellent use-case for such paper, the authors should choose whether they are writing “about COVID–19” or “about computational epidemiology, using COVID–19” as an example”.

Right in the introduction, the authors jump from historical epidemiological modeling, mentioning work by Kermack & McKendrick (L50), to very restrictive comments about “government intervention measures and the development of vaccines” (L56) and “developing vaccine strategies” (L59). Vaccines and (global) government-enforced control measures are not a common thing in the field of infectious diseases and control measures, when they exist, often rely on community-driven interventions (e.g. for infectious diseases, NTDs…) or from human behavior. The authors should consider extensive modifications to better reflect that they developed a framework to investigate the COVID–19 outbreak in different regions/countries, therefore justifying the discussions about vaccines and government-enforced control measures.

2) Proper definition of R_t

In the introduction, R_t is defined as “the average number of secondary cases that could be generated by a primary case, if the transmission dynamics remained the same thereafter time t. It is an essential metric to indicate the trajectory of prevalence[10]” (L60–63).

There is likely a confusion here between the *instantaneous reproduction number* R_t and the *effective average reproduction number* R_{eff}. R_t does not make assumptions about a stationary state to predict the number of upcoming incident cases, but is defined exactly as the average number of incident cases spanning from all currently infected cases at time t. It is merely the result of all interactions between susceptible and infectious individuals at time t. It is time-varying by definition (see e.g. https://doi.org/10.1093/aje/kwh255 and https://doi.org/10.1186/1472-6947-12-147).

3) Absence of internal validation and single choice for external validation

As a sound approach, the authors first demonstrate that under controlled assumptions, DARt successfully estimates Rt and incident cases in Fig 3B. While I am “graphically” convinced, the

manuscript lacks at least a table to report numerically on how DARt (and EpiEstim) deviate from the true R_t and incidence series.

As external validation, the authors decided to only investigate EpiEstim. What led to that choice ? There are other powerful tools available that could have been used in this benchmark, among which epitools and epidemia (also implementing Bayesian methods).

4) **Possible over-interpretation of model fits**

In Figure 3, the authors demonstrate that DARt very accurately captures the epidemic dynamic for R_t (3B), incident cases J_t (3C), reported cases C_t (3D).

I am concerned that some of the improvement described by the author may be the result of overfitting at the edge of the incidence curves. In a transparent effort to make replicable examples available (which is very appreciated), the authors provide the code that was used for the simulation experiements on their GitHub repository (https://github.com/Kerr93/DARt/blob/master/code_ocean/Simulation-exp.html where Fig 3B is likely a modified version of output from code block `In [44]` from the above notebook). It should be noted that in code black `In [5]`, the author padded the incidence vectors at both extremities with constant values for a length that is equal to the length of the generation time distribution. Any estimation method not taking into account this artificial padding would result in overly large estimates of R_t, rapidly coming down to more realistic values. This is exactly what can be observed from EpiEstim results in Fig 3B up until t = 8 days (?), which coincides with the first value not padded in the vector, resulting from epidemic spread. One should also note that while DARt seemingly outperforms EpiEstim at the edges of the curves, the credible intervals denote much larger uncertainty at these locations.

Another concern is the direct comparison between EpiEstim results and DARt results : DARt formerly accounts for observation delays (parameterized) and under-reporting. In Figure 4 and its legend, the authors conclude that “CIs for Rt from EpiEstim are quite narrow (even cannot be easily observed from this figure), indicating the underestimated uncertainty of estimation” (L389–391).
First, the agreement between values from EpiEstim is striking in all 4 panels, with exception of “hiccups” that are not replicated by DARt. These are likely caused by very briefs increase in cases, that backward smoothing successfully removes.
Second, it is incorrect to state that uncertainty is “underestimated” by EpiEstim, which makes the underlying hypothesis that data is fully observed. The apparent increase in uncertainty seen with DARt is at least the result of the observation process, that introduces a new layer of uncertainty.
Third, as external validation of the model predictions, the dates predicted to have high values of $Pr(M_t = 1)$ should be summarized in a separate table and corresponding events in the studied regions/countries be indicated. One would hope that most dates would coincide or be close to major changes in control policies.

As another example of over-interpretation of DARt improvements, the authors state at L458–461 that the periodic pattern observed in Sweden (with obvious reporting issues) had been smoothed by DARt. In fact, it had also been largely smoothed by EpiEstim (Fig 4D). Upon close

data inspection, Sweden seems to have a reporting bias with many under-reported cases on Tuesdays and Wednesdays, and corresponding over-reporting the following Thursdays and Fridays. This is a common issue with national reporting, occurring in other countries (e.g. France : https://covid19.who.int/region/euro/country/fr (use daily view)), where under-reporting is quickly compensated over the next working days. The smoothing here is consistent with a weekly rolling average. This is mentioned again L490–492 and the periodicity of the over/under reporting should immediately point towards a 7-day sliding window, rather than exploring different values : Fig 6B and 6C are unnecessarily and proper data analysis should first investigate the periodicity of misreporting.

5) Discuss the implications of smoothing estimates on data interpretation

At L411–414, the authors explain that DARt is unaffected by “major correction in reporting standard”. One should be very cautious as to how this would generalize to other outbreaks: in some settings, localized super-spreading events (e.g. in Althaus https://doi.org/10.1016/S1473-3099(15)70135-0) are of epidemiological interest: for these occasions, quick bursts for R_t *are* expected. There are two stories here: the overall populational approach (presented here) and the localized one. Both are relevant and DARt not replicating such quick bursts should at least be discussed.

In fact, it’s important to discuss when smoothing should be applied: noisy input data are expected to result in noisy outputs and pre-processing may be required. This is merely an issue of phrasing, however.

Minor comments

- Overall comment: please shorten all figure legends so that they not include interpretation of result but merely describe what the graph presents
- Overall comment: avoid use of vague expressions such as “to a greater of lesser extent”
- Overall comment: the English level is notably inconsistent across the manuscript ; please harmonize
- Overall comment: harmonize the labelling of references throughout the text (use author names instead of generic Ref[n] ?)
- Overall comment: Use CI for “Confidence Intervals” and CrI for “Credibility Intervals” (e.g. L132), as both are not completely equivalent
- Overall comment: The results section contains a lot of statements that could be moved to the discussion to help the reader distinguish between the findings and the interpretation the authors make of these. Should results and discussion be combined, consider reorganizing the manuscript to discard the “Discussion and conclusion” section (L519+)
- L26: “uncertainty” *per se* is not an issue, rather, its proper quantification is
- L53–54: stating that biological features of a virus are stationary is an overstatement, that the current context contradicts (e.g. the large variation in transmissibility of the different COVID–19 VOC)
- L107: “not accurate” should be changed to “inaccurate”; “blurred” does not sound like the proper term here

- L146–152: consider re-writing and avoid repetitive use of the words “observation” and “process”
- L152–156: avoid use of such generalizing statements that are not useful for the reader. For example, financial analysis has been notoriously using MCMC to study stochastic volatility (+L154: extra unnecessary comma)
- L156: Figure 7 is referenced in the legend for Figure 1. Either Figure 7 should become Figure 2 or be combined with Figure 1 so that a single figure be used instead
- L183: a verb is missing “backward smoothing is to compute”
- L232 : Correct “capturing” to “capture”
- L404: avoid ambiguous statements like “around this time” and indicate the exact lockdown date in Wuhan (January 23rd)
- L455: indicate proper labels for the mentioned VOC (e.g. South-African : B.1.351 or Beta, etc.)
- L214–216: provide reference
- L586: use conventional abbreviation “etc.”
- L577–599: I am unconvinced by how far the authors assume “one” could develop their method by incorporating features usually found in individual/agent-based models.
- L668–669: provide reference for the statement that “R_t is commonly assumed to follow a Gaussian random walk”
- L677: what is the proposed value for Delta ?
- L728 (and others): consider use of large brackets if using the Microsoft Office equation editor, so that grouping remains intelligible
- Reference 4 makes incorrect use of full authors first name and abbreviated last names
