# Supplementary material for: Bayesian data assimilation for estimating instantaneous reproduction numbers during epidemics: Applications to COVID-19
Source: PLoS Comput Biol. 2022 Feb 23;18(2):e1009807. doi: 10.1371/journal.pcbi.1009807 (PMC8923496; doi:10.1371/journal.pcbi.1009807)
Supplement: Attachment — Submitted filename: Yang_et_al_review_R1.pdf [file pcbi.1009807.s004.pdf]

The authors of the manuscript entitled “Bayesian data assimilation for estimating transmission dynamics in computational epidemiology” provided point-by-point responses to the comments raised in my first review.

I am overall convinced by many of the modifications the authors have made and am thankful for the very hard work they have provided, especially by including another estimation method in their benchmark. I appreciate fully how time-consuming such modifications can be and am impressed by the time and effort put into improving this manuscript. **Overall, this work has substantially improved and I have a few more follow-up points to raise, related to my original major comment #4 about possible over-interpretation of model results.**

In the following, the majors points I raised during my first review will only be retained with their title, and appended with the author response, followed by my new comments, should their be any. The line numbers, when specified, are in reference to the track-changes version of the manuscript.

Major comments

1) **Inconsistency between manuscript title and overall content**

[Response]: We agree with the reviewer. We have modified the manuscript title to “Bayesian data assimilation for estimating epidemic evolution: a COVID-19 study” to make the scope more focused and clear.

The new title makes the scope of the study clearer.

[Response]: Thanks for the suggestion. We have rewritten the beginning part in the introduction. Now it focuses on the motivation of estimating the time-varying epidemic model parameters.

The modifications in the introduction have preserved the 3 points of interest to the authors (lagging, averaging, uncertainty) and reads nicely, albeit for some typos (L155 “impact**s**”, L200 “~~the~~ rapidly changing”, L209 “arrival” -> “availability”, L215 “estimate**s**”, L218 “come**s**”).

It is now clearer in the manuscript that the object of this work is to tackle the rapid spread of an emerging infectious disease, in a context of rapidly evolving control measures by policymakers. In this respect, the changes address my initial comment and I am satisfied by these.

2) **Proper definition of R_t**

[Response]: Thanks for pointing out the difference between the instantaneous reproduction number Rt and the effective average reproduction number Reff. We agree with the reviewer that the Rt is time- varying by definition. We have rephrased the definition of Rt following the convention (https://doi.org/10.1371/journal.pcbi.1008409, Figure 1) “More formally, the instantaneous reproductive number is defined as the expected number of secondary

infections occurring at time t, divided by the number of infected individuals, each scaled by their relative infectiousness at time t (an individual's relative infectiousness is based on the generation interval and time since infection)." We note that this definition is also consistent with the Rt definition in the popular package 'EpiEstim'.

The authors have addressed my concern about correctly defining Rt throughout the manuscript

3) Absence of internal validation and single choice for external validation

[Response]: Thanks for the suggestion. We have added a table (Supplementary Table 2) to better demonstrate the quantitative evaluation. We can observe that DARt smoothing has greatly contributed to uncertainty reduction.

Supplementary Table 2 addresses my concern by providing the means and standard deviations of Rt and Jt (incidence series).

[Comment]: Could the author explain why Supplementary Table 2 doesn't have estimates for Jt with EpiEstim? If these can be calculated, they should be included.

[Response]: Thanks for the suggestion. We used the EpiEstim as the baseline considering its popularity in the literature. Following the reviewer's suggestion, we have included another Bayesian baseline - EpiNow (version 2, reflecting the latest development) for comparison in the revised man uscript.

I am satisfied with including EpiNow2, which was suggested by Reviewer #2.

4) Possible over-interpretation of model fits

[Response]: Thanks for the comments. We have changed the way of generating the infected curve j_t, which is initialized to be 1. Please refer to the updated "Validation through simulation" section.

For this specific answer, the authors now detail the simulation process that generates the "Synthesized Rt" curves shown in Figure 3. My original concern was that the poor performance of EpiEstim at the edges of Fig 3B could be the artificial result of feeding EpiEstim with incorrect, non-existing data at the edges of the curve. Upon the revisions provided by the authors especially in Fig 3B, it seems this intuition was right. The revised figure now makes clear that the benefits of the present developments arise from the state-transition model, notably better at capturing abrupt changes. This better performance is "by design" since the proposed model explicitely allows for abrupt changes, a feature that is not often developed in epidemiological modelling.

[Comment #1]: The Python notebook in the GitHub repository has not been updated to reflect the changes in how Jt is generated (Code block `In [5]`). This should be done to avoid misleading users who would not be auditing the code and wrongly assume that EpiEstim is vastly outperformed when it actually isn't.

[Comment #2]: In their revision, the authors have modified the x-axis scales in Fig 3 (in accordance with more data being now available in worldwide COVID time-series? Or is that a pure artificial attempt at showing multiple scenarios, with a first outbreak followed by two abrupt decreases in transmission (e.g. curfew then lockdown) and an abrupt increase weeks later e.g. abandon of control measures?).
The panels are however not all consistent with each other, one showing curves for DARt, EpiEstim and EpiNow2 (3B), while some others only show DARt and EpiNow (3D, 3E). I believe all three benchmarked methods should present equally their performance. Is there a particular reason the authors decided to drop EpiEstim curves from panels 3D and 3E? These were available pre-revision respectively in panels 3B and 3C.

[Comment #3]: In their revised manuscript, the authors state that:

- L493–494: (paraphrased) "piNow Jt does not align nicely with the simulated curve". This is a bit of an overstatement: rather, EpiNow Jt demonstrates that it is somewhat "over-smoothed". In particular, I would be interested to see the cumulated curves corresponding to these time-series: I am reasonably sure that the total cumulative cases would be near identical, with the difference in peak "width" (wider for EpiNow) possibly compensating for its underestimation of point-estimate values
- L496–498: this time, the authors rightly point that DARt outperforms EpiNow2. It should still be noted that EpiNow2 point-estimates seem to fall within DARt 95%-CrI.

Overall, I believe the message that DARt outperforms other methods should still be toned down, in favor of its clear ability to detect abrupt changes in transmission.

[Response]: We agree with the reviewer. We have rewritten the previous statement to make the comparison more objective. Also, we give more details about the sharp changing points.

Overall, the change in phrasing (especially in the legend for Fig 4) addresses my comment. I thank the authors for making Fig 4 easier to read, by splitting it into 3*4 panels instead of 2*4. Having the "observed" data (*i.e.,* the closest that can be assumed to be the "ground truth") as overlayed bar also helps the reader in distinguishing between predictions (lines and observations (bars).

[Comment #1]: Comparing the two versions of Fig 4 (before/after revision), I note that the x-axis have been adjusted for all 4 locations (likely resulting for more data being available worldwide):

- Wuhan: 16/12 to 02/03 became 20/12 to 26/02. The change here is hardly noticeable in terms of data.
- Hong Kong: 03/07 to 04/12 became 14/11 to 13/03. The dropped period seemed to consist in a high transmission period. Why was that period discarded?
- UK: 09/03 to 05/04 became 26/12 to 21/08 hence now including the 1st wave. It feels sensible to **add** more data to benchmark DARt rather than remove some as is the case for Hong Kong
- Sweden: 07/03 to 12/12 became 06/01 to 01/09. I can't entirely make sense of this change.

I would appreciate if the authors could clarify why these data periods have been changed between initial submission and the first revision, as well as confirm the validity of the x-axis scale for each panel (esp. Sweden, the scales before and after revision don't seem to match).

[Comment #2]: The authors have now added in the text the dates at which P(Mt=1) are predicted to spike, along with corresponding events by policymakers. It's satisfying to see that the state-transition model nicely captures these events.

On a personal note, it is enjoyable, yet frightening, to see that "obvious epidemiological expectations" are met with Euro 2020 causing sustained increases in transmission. It's also reassuring to see that easing control measures apparently happened at a time where mass vaccination was efficient enough to keep the epidemic somewhat under control.

[Response]: We agree with the reviewer that the best practice using EpiEstim includes data smoothing with the periodicity of misreporting because EpiEstim does not consider the observation uncertainty. One advantage of the proposed DARt is the ability to quantify the observation uncertainty and smoothing the data automatically in the inference process. We have removed Fig 6B and 6C which are unnecessary as suggested by reviewers.

I thank the authors for acknowledging such good practice in a data-driven approach. While DARt provides a more versatile framework compared to window-based methods, no modeler/analyst should ever process data as that from Sweden as raw input.

5) **Discuss the implications of smoothing estimates on data interpretation**

[Response]: In the case of miss-reporting, there is usually a sharp increase in Ct and then immediate decrease on the next day. In the case of super-spreading, usually a sharp increase in Ct is followed by the increasing values of Ct in the following days. Our model can automatically be adapted to these different scenarios. In the simulation experiment, we have examined the scenario that Rt experiences a sharp increase which could be caused by super-spreading. In real-world experiments, we can return a rather smooth Rt curve for Sweden which has significant mis-reporting pattern.

The authors make a fair point in exploring the implications of misreporting (*i.e.* very localized outburst in Ct) and super-spreading (*i.e.* explosion of cases, followed by less dramatic transmission). The comparison between DARt results from real-world data (e.g. misreporting in Sweden) and their simulations (sharp increases that could be somewhat equivalent to super-spreading) is convincing enough to me.

Minor comments

All the minor comments I had raised are sufficiently addressed. There are still some grammatical/spelling issues that would warrant a careful reading, but this is minor at this point.
