# Supplementary material for: Bayesian data assimilation for estimating instantaneous reproduction numbers during epidemics: Applications to COVID-19
Source: PLoS Comput Biol. 2022 Feb 23;18(2):e1009807. doi: 10.1371/journal.pcbi.1009807 (PMC8923496; doi:10.1371/journal.pcbi.1009807)
Supplement: Attachment — Submitted filename: response_to_reviewers.docx [file pcbi.1009807.s003.docx]

Dear editor

Here, we provide our responses to the review comments and descriptions of the changes we have made in the manuscript.

**Reviewer #1:**

In the manuscript entitled “Bayesian data assimilation for estimating transmission dynamics in computational epidemiology”, the authors present an analytical framework that aims at addressing several common issues that epidemiologists face in the context of an epidemic outbreak. During such times, several metrics are used to characterize the spread of a pathogen. Rt, the “time-varying reproduction number”, is one of them and can be defined as the average number of secondary cases spanning from an index case occurring at time t. The variations of Rt reflect directly on how aggressive the epidemic is at a given time step, and this measure is often used by policymakers to implement or alleviate control measures, as well as to assess their impact.

The manuscript provides a powerful new tool to the existing literature on estimating parameters of interest to assess epidemic outbreaks, with the added benefit of a public GitHub repository providing reproducible examples. I am overall convinced that this work could be published in PLoS Computational Biology, but major changes are necessary before acceptance.

As an outbreak unfolds, more and more data become available both with respect to the pathogen biology / life cycle / disease natural history, as well epidemic spread. These two elements pave the way towards building an epidemic model.

As a highlight, the authors identify three main sources of biases in currently available methods:

1) Lagging: incident cases occurring at time t may not be observed immediately but after a random delay

2) Averaging inference: the estimated parameters are assumed constant within estimation windows

3) Uncertainty

The main addition of this work to the current epidemiologist toolbox is the methodological development around the forward estimation and backward smoothing of prior estimates as data accumulates. As a first proof of concept, the authors simulated controlled epidemic processes governed by calibrated values of Rt and compared the performance of their DARt method with that from EpiEstim (Cori et al.). After this simulated validation, they set out to assess the performance of DARt with COVID–19 incidence data from 4 regions/countries: Wuhan, Hong Kong, the United Kingdom and Sweden.

We would like to thank the reviewer for the constructive comments and acknowledging the methodological novelty of our work. Detailed point-to-point responses are listed below.

The writing should be heavily improved to i) ease readability and ii) clarify the scope of the work.

**[Response]:** Thanks for the suggestion. We have removed some redundant contents in the main text and improved the English writing quality. We believe it is easier for the reader to read now.

Furthermore, while it provides undeniable methodological development, the statements about overall reduced uncertainty should be toned done to better reflect that uncertainty is better split between its main sources, and subsequently propagated into posterior distributions. In particular, the overall process of estimating reproduction numbers still makes use of the commonly reported generation time distribution approximated by serial interval; parametric or empirically-calibrated reporting delay as well as incubation time; underlying S(E)IR structure…

**[Response]:** We agree with the reviewer. We split the sources contributing to the uncertainty of time-varying estimates and focus on quantifying and reducing the uncertainties from observations (e.g. reported numbers) rather than the distributions of time delays. Our revisions include: 1) We have integrated the above statements into the introduction; 2) Although we mainly focus on uncertainties in observations, we add some sensitivity analyses (in the supplementary document) to show the performance of our model in response to different settings of distributions of time delays.

Last but not least, I am somewhat disturbed by some phrasing used to benchmark the performances of DARt against EpiEstim in the controlled simulations: the (overly-long) legend for Figure 3 reads more as a negative critic towards EpiEstim where it should only emphasize that methodological developments helped at better capturing abrupt changes in epidemic process. This comment applies all throughout the text and I strongly suggest that previous works, conducted by different teams, be presented in a more respectful way.

**[Response]:** Thanks for the suggestion. We have rewritten and shorten the legends. The writing is reorganised in a more objective and respectful way.

**Major comments**

1) Inconsistency between manuscript title and overall content

The title misleads the reader into expecting the description of a general Bayesian framework for computational epidemiology. Instead, the manuscript focuses entirely on the COVID–19 outbreak that has kept every epidemiologist busy over the past ~1.5 year. While I obviously agree that in the present global sanitary context, COVID–19 is the leading cause of epidemiological model development and an excellent use-case for such paper, the authors should choose whether they are writing “about COVID–19” or “about computational epidemiology, using COVID–19” as an example”.

**[Response]:** We agree with the reviewer. We have modified the manuscript title to “Bayesian data assimilation for estimating epidemic evolution: a COVID-19 study” to make the scope more focused and clear.

Right in the introduction, the authors jump from historical epidemiological modeling, mentioning work by Kermack & McKendrick (L50), to very restrictive comments about “government intervention measures and the development of vaccines” (L56) and “developing vaccine strategies” (L59). Vaccines and (global) government-enforced control measures are. not a common thing in the field of infectious diseases and control measures, when they exist, often rely on community-driven interventions (e.g. for infectious diseases, NTDs…) or from human behavior. The authors should consider extensive modifications to better reflect that they developed a framework to investigate the COVID–19 outbreak in different regions/countries, therefore justifying the discussions about vaccines and government-enforced control measures.

**[Response]:** Thanks for the suggestion. We have rewritten the beginning part in the introduction. Now it focuses on the motivation of estimating the time-varying epidemic model parameters.

2) Proper definition of Rt

In the introduction, Rt is defined as “the average number of secondary cases that could be generated by a primary case, if the transmission dynamics remained the same thereafter time 𝑡. It is an essential metric to indicate the trajectory of prevalence[10]” (L60–63).

There is likely a confusion here between the instantaneous reproduction number Rt and the effective average reproduction number Reff. Rt does not make assumptions about a stationary state to predict the number of upcoming incident cases, but is defined exactly as the average number of incident cases spanning from all currently infected cases at time t. It is merely the result of all interactions between susceptible and infectious individuals at time t. It is time-varying by definition (see e.g. https://doi.org/10.1093/aje/kwh255 and <https://doi.org/10.1186/1472-6947-12-147>).

**[Response]:** Thanks for pointing out the difference between the instantaneous reproduction number Rt and the effective average reproduction number Reff. We agree with the reviewer that the Rt is time-varying by definition. We have rephrased the definition of Rt following the convention (<https://doi.org/10.1371/journal.pcbi.1008409>, Figure 1) “More formally, the instantaneous reproductive number is defined as the expected number of secondary infections occurring at time t, divided by the number of infected individuals, each scaled by their relative infectiousness at time t (an individual’s relative infectiousness is based on the generation interval and time since infection).” We note that this definition is also consistent with the Rt definition in the popular package ‘EpiEstim’.

3) Absence of internal validation and single choice for external validation

As a sound approach, the authors first demonstrate that under controlled assumptions, DARt successfully estimates Rt and incident cases in Fig 3B. While I am “graphically” convinced, the manuscript lacks at least a table to report numerically on how DARt (and EpiEstim) deviate from the true Rt and incidence series.

**[Response]:** Thanks for the suggestion. We have added a table (Supplementary Table 2) to better demonstrate the quantitative evaluation. We can observe that DARt smoothing has greatly contributed to uncertainty reduction.

As external validation, the authors decided to only investigate EpiEstim. What led to that choice? There are other powerful tools available that could have been used in this benchmark, among which epitools and epidemia (also implementing Bayesian methods).

**[Response]:** Thanks for the suggestion. We used the EpiEstim as the baseline considering its popularity in the literature. Following the reviewer’s suggestion, we have included another Bayesian baseline - EpiNow (version 2, reflecting the latest development) for comparison in the revised manuscript.

4) Possible over-interpretation of model fits

In Figure 3, the authors demonstrate that DARt very accurately captures the epidemic dynamic for Rt (3B), incident cases Jt (3C), reported cases Ct (3D). I am concerned that some of the improvement described by the author may be the result of overfitting at the edge of the incidence curves. In a transparent effort to make replicable examples available (which is very appreciated), the authors provide the code that was used for the simulation experiments on their GitHub repository (https://github.com/Kerr93/DARt/blob/master/code_ocean/Simulation-exp.html ) where Fig 3B is likely a modified version of output from code block In [44] from the above notebook). It should be noted that in code black In [5], the author padded the incidence vectors at both extremities with constant values for a length that is equal to the length of the generation time distribution. Any estimation method not taking into account this artificial padding would result in overly large estimates of Rt, rapidly coming down to more realistic values. This is exactly what can be observed from EpiEstim results in Fig 3B up until t = 8 days (?), which coincides with the first value not padded in the vector, resulting from epidemic spread. One should also note that while DARt seemingly outperforms EpiEstim at the edges of the curves, the credible intervals denote much larger uncertainty at these locations.

**[Response]:** Thanks for the comments. We have changed the way of generating the infected curve $j_{t}$ , which is initialized to be 1. Please refer to the updated “Validation through simulation” section.

Another concern is the direct comparison between EpiEstim results and DARt results: DARt formerly accounts for observation delays (parameterized) and under-reporting. In Figure 4 and its legend, the authors conclude that “CIs for Rt from EpiEstim are quite narrow (even cannot be easily observed from this figure), indicating the underestimated uncertainty of estimation” (L389–391). First, the agreement between values from EpiEstim is striking in all 4 panels, with exception of “hiccups” that are not replicated by DARt. These are likely caused by very briefs increase in cases, that backward smoothing successfully removes. Second, it is incorrect to state that uncertainty is “underestimated” by EpiEstim, which makes the underlying hypothesis that data is fully observed. The apparent increase in uncertainty seen with DARt is at least the result of the observation process, that introduces a new layer of uncertainty. Third, as external validation of the model predictions, the dates predicted to have high values of Pr(Mt = 1) should be summarized in a separate table and corresponding events in the studied regions/countries be indicated. One would hope that most dates would coincide or be close to major changes in control policies.

**[Response]:** We agree with the reviewer. We have rewritten the previous statement to make the comparison more objective. Also, we give more details about the sharp changing points.

As another example of over-interpretation of DARt improvements, the authors state at L458– 461 that the periodic pattern observed in Sweden (with obvious reporting issues) had been smoothed by DARt. In fact, it had also been largely smoothed by EpiEstim (Fig 4D). Upon close data inspection, Sweden seems to have a reporting bias with many under-reported cases on Tuesdays and Wednesdays, and corresponding over-reporting the following Thursdays and Fridays. This is a common issue with national reporting, occurring in other countries (e.g. France: https://covid19.who.int/region/euro/country/fr (use daily view)), where underreporting is quickly compensated over the next working days. The smoothing here is consistent with a weekly rolling average. This is mentioned again L490–492 and the periodicity of the over/under reporting should immediately point towards a 7-day sliding window, rather than exploring different values: Fig 6B and 6C are unnecessarily and proper data analysis should first investigate the periodicity of misreporting.

**[Response]:** We agree with the reviewer that the best practice using EpiEstim includes data smoothing with the periodicity of misreporting because EpiEstim does not consider the observation uncertainty. One advantage of the proposed DARt is the ability to quantify the observation uncertainty and smoothing the data automatically in the inference process. We have removed Fig 6B and 6C which are unnecessary as suggested by reviewers.

5) Discuss the implications of smoothing estimates on data interpretation

At L411–414, the authors explain that DARt is unaffected by “major correction in reporting standard”. One should be very cautious as to how this would generalize to other outbreaks: in some settings, localized super-spreading events (e.g. in Althaus https://doi.org/10.1016/S1473-3099(15)70135-0) are of epidemiological interest: for these occasions, quick bursts for Rt are expected. There are two stories here: the overall populational approach (presented here) and the localized one. Both are relevant and DARt not replicating such quick bursts should at least be discussed. In fact, it’s important to discuss when smoothing should be applied: noisy input data are expected to result in noisy outputs and pre-processing may be required. This is merely an issue of phrasing, however.

**[Response]:** In the case of miss-reporting, there is usually a sharp increase in Ct and then immediate decrease on the next day. In the case of super-spreading, usually a sharp increase in Ct is followed by the increasing values of Ct in the following days. Our model can automatically be adapted to these different scenarios. In the simulation experiment, we have examined the scenario that Rt experiences a sharp increase which could be caused by super-spreading. In real-world experiments, we can return a rather smooth Rt curve for Sweden which has significant mis-reporting pattern.

**Minor comments:**

- Overall comment: please shorten all figure legends so that they not include interpretation of result but merely describe what the graph presents

[**Response**] Following the reviewer’s suggestion, we have shorten the redundant contents in the figure legends.

- Overall comment: avoid use of vague expressions such as “to a greater of lesser extent”

[**Response**]: We have revised it.

- Overall comment: the English level is notably inconsistent across the manuscript; please harmonize

[**Response**]: We have improved it.

- Overall comment: harmonize the labelling of references throughout the text (use author names instead of generic Ref[n] ?)

[**Response**] Thanks for the suggestion. We have carefully checked and harmonised the writing and reference.

- Overall comment: Use CI for “Confidence Intervals” and CrI for “Credibility Intervals” (e.g. L132), as both are not completely equivalent

[**Response**] Thanks for pointing this out. We use the Credibility Intervals for Bayesian statistics. Now the abbreviations are corrected.

- Overall comment: The results section contains a lot of statements that could be moved to the discussion to help the reader distinguish between the findings and the interpretation the authors make of these. Should results and discussion be combined, consider reorganizing the manuscript to discard the “Discussion and conclusion” section (L519+)

[**Response**] We agree with the reviewer. The results section has been reorganised.

- L26: “uncertainty” per se is not an issue, rather, its proper quantification is

**[Response]** Thanks for the suggestion. We have rephrased the description.

- L53–54: stating that biological features of a virus are stationary is an overstatement, that the current context contradicts (e.g. the large variation in transmissibility of the different COVID–19 VOC)

**[Response]** Thanks for the suggestion. We have rephrased this sentence.

- L107: “not accurate” should be changed to “inaccurate”; “blurred” does not sound like the proper term here

**[Response]** Thanks for the suggestion. We have rephrased this sentence.

- L146–152: consider re-writing and avoid repetitive use of the words “observation” and “process”

**[Response]** Thanks for the suggestion. We have rephrased this sentence.

- L152–156: avoid use of such generalizing statements that are not useful for the reader. For example, financial analysis has been notoriously using MCMC to study stochastic volatility (+L154: extra unnecessary comma)

**[Response]** Thanks for the suggestion. We have deleted that statement to make the study more focused.

- L156: Figure 7 is referenced in the legend for Figure 1. Either Figure 7 should become Figure 2 or be combined with Figure 1 so that a single figure be used instead

**[Response]** changed

- L183: a verb is missing “backward smoothing is to compute”

**[Response]** updated

- L232 : Correct “capturing” to “capture”

**[Response]** updated

- L404: avoid ambiguous statements like “around this time” and indicate the exact lockdown date in Wuhan (January 23rd)

**[Response]** updated

- L455: indicate proper labels for the mentioned VOC (e.g. South-African: B.1.351 or Beta, etc.)

**[Response]** updated

- L214–216: provide reference

**[Response]** updated

- L586: use conventional abbreviation “etc.”

**[Response]** updated

- L577–599: I am unconvinced by how far the authors assume “one” could develop their method by incorporating features usually found in individual/agent-based models.

**[Response]** Thanks for the comments. We have removed these statements.

- L668–669: provide reference for the statement that “Rt is commonly assumed to follow a Gaussian random walk”

**[Response]** updated

- L677: what is the proposed value for Delta?

**[Response]** The detailed descriptions of experimental settings can be found in the supplementary material.

- L728 (and others): consider use of large brackets if using the Microsoft Office equation editor, so that grouping remains intelligible

- Reference 4 makes incorrect use of full authors first name and abbreviated last names

**[Response]** updated

**Reviewer #2:**

A summary of my comments (detailed below): The method described in this paper is interesting and promising, given its success at handling the issues it sets out to address (lags, uncertainty and averaging), and the demonstrated comparisons to results output from EpiEstim. It is necessary to add a thorough comparison to EpiNow. The deconvolution discussion and comparisons should be extended, and the accuracy of Supplementary Figure 3 should be carefully checked (and commented on, depending on the results of the check). This paper needs to consider uncertainty from potential misspecification of the generation interval distribution and the observation delay distribution. The analysis of the sensitivity of the method to truncation needs to be separate from the issue of adding variance to the estimates of the parameters for the generation interval distribution and observation delay distribution. Some issues with writing, organization, and figures should be fixed.

**[Response]:** All these comments here have been expanded in the later part of this letter. We add responses in the following paragraphs one by one.

The instantaneous reproductive number Rt has become a ubiquitous tool for assessing the state of the COVID-19 pandemic in different regions across the globe. It has been used to inform policy decisions such as whether to impose or lift restrictions like lockdowns and mask mandates, and to determine the effect of such interventions.

Yet computing Rt from the data that is typically available to researchers during the course of the pandemic is nontrivial because of the forward-looking nature of the interpretation of Rt as the average number of secondary infections a person who is infectious at time t will produce in the next timestep, a measure which approximates the number of secondary infections a person infected at time t will produce over the course of their infection using only data accessible up to time t. Moreover, Rt computations depend on epidemiological parameters that are often unknown or even unknowable in real analyses.

Although best practices for computing Rt have been proposed since the beginning of the pandemic (Gostic et all 2020, e.g.), several problems have remained as important challenges to computing Rt accurately. These include the lag between time of infection and measured case numbers from testing, hospitalization, or deaths; the difficulty in properly accounting for multiple sources of uncertainty; and the ambiguity in selecting a window size for averaging in computing Rt using what are currently state of the art methods.

This paper advances the current state of the field by proposing a novel Bayesian method, DARt, for addressing these three problems using the technique of data assimilation. It uses simulated data to demonstrate advantages of this method compared to methods for computing Rt using the package EpiEstim, which is currently a widely used, recommended method for computing Rt in real applications. One particularly interesting feature of the method is how it handles abrupt changes in Rt through the incorporation of a binary latent variable that infers the probability of large changes in Rt in short periods of time.

This paper is not the first, however, to implement a Bayesian method with the goal of addressing lagged data, uncertainty and averaging in computations of Rt. In particular, the package EpiNow (and its more recent iteration EpiNow2), developed by Abbott et al 2020, implements another Bayesian method meant to to solve these issues within the context of best practices for computing Rt. While the authors of this paper briefly mention EpiNow2, they do not compare their methods for inferring the curve of incident infections from the curve of observations and Rt to the methods used by EpiNow2 in detail. Given the importance of EpiNow2 as a highly developed tool for solving problems this paper is addressing as well, the authors’ choice to compare only to EpiEstim and not to EpiNow2 is a major oversight that prevents the researchers from putting their work in context with currently available tools.

**[Response]:** Thanks for the advice. We have included EpiNow2 as a comparative method both in the simulation experiments and real-world application.

The paper also deemphasizes the importance of the Goldstein et al deconvolution method for taking into account lags in Rt calculations, comparing DARt to deconvolution only in section 2.3 of the supplementary information. Deconvolution has the serious disadvantages of not allowing for computation of uncertainty in the inference of the incident infection curve and requiring a window size choice in its calculation of Rt, but it is typically very successful at producing a point estimate of the infection curve from the curve of observations when the distributions of generation interval and delays to observation are assumed to be known and with an appropriate choice of stopping criterion for the Richardson-Lucy algorithm.

The lack of agreement between the simulated infection curve and the deconvolved infection curve in Supplementary Figure 3 is surprising and may indicate an issue with the implementation of the deconvolution code. Such an issue can occur if the indexing of time in the delay distribution is off compared to the indexing of time in the vectorized curve of observations (one must be careful to keep track of whether the delay distribution before truncation mathematically begins with the probability that the delay equals 0 days vs. the delay equaling 1 day; such an off-by-one error can cause disagreements like the one shown here - in particular, the disagreement between the location of the peak of the deconvolved curve and the true infection curve - although it is not the only possible issue). This disagreement should be carefully checked; if the deconvolution is truly not reproducing the peak correctly, this requires at least a comment on the intuition for why this is happening.

**[Response]:** Thanks for pointing out this issue and detailed explanations. Instead of using the R-L algorithm, we apply the recent implementation of deconvolution from EpiNow2 (i.e. the ‘estimate_infections’ function). The results are shown in Supplementary Figure 3.

This paper also neglects a source of uncertainty that is very important to real-life calculations of Rt. Namely, the observation delay distribution is very rarely known exactly (or even very accurately at all); even the generation interval distribution can be unknown, for example in the case of new COVID-19 variants. The authors provide a sensitivity analysis in supplementary figure 5 that is meant to address this issue, but the analysis is insufficient for a few reasons. First, it seems to test the effects of different thresholds for the truncation and samples from generation time distribution and observation delay distribution with variance in the parameters in a single plot. Second, there is no demonstrated analysis of the sensitivity of the method to cases where the shape of the assumed generation interval and/or observation delay are misspecified (with the wrong shape/scale/logmean/variance compared to the generation interval and observation delay used to generate the observation curve). Such misspecification can, at least for some methods, severely impact the accuracy of the inferred infection and Rt curves. Ideally, a method for inferring the infection curve and Rt with uncertainty would take into account uncertainty derived from the likely misspecification of generation interval and observation delay distribution in real applications; barring this, which is difficult to model, the sensitivity analysis should at least be modified to: a) test the effects of delay distribution minimum probability threshold and uncertainty in the delay distributions separately, and b) test the sensitivity of the method to cases where the delay distributions are misspecified, not just uncertain.
**[Response]:** Thanks for the advice. In the aspect of reducing uncertainty, our model mainly focuses on investigating and reducing uncertainties resulted from reported numbers (observations) using smoothing. However, when the delay distribution has uncertainties, we can sample multiple distributions from which $R_{t}$ results considering uncertainties in delays can be generated. In Supplementary section 6 Sensitivity Analysis, we add sensitivity analysis in respect to different distributions of time delays and truncation threshold.

There are a few more minor issues that need to be addressed. In no particular order:
There are some scattered language and grammatical errors which need to be edited.
The arbitrary choice of Delta in the prior for Mt is not ideal, especially since the decision to allow Rt to increase discontinuously for the Hong Kong analysis (and the constraint that Rt not increase discontinuously in other cases) is inserted “by hand” rather than “discovered” by means of the inference.

**[Response]:** Thanks for pointing out this problem. We have rerun the Hong Kong experiment using the same settings as other regions, where detailed descriptions of experimental settings can be found in the supplementary document.

The extended description of the algorithm in the “Results” section seems potentially misplaced; I would suggest considering making this discussion much more concise and putting more of these details in the Methods section.

**[**Response]: Thanks for the suggestion. We have moved the description of the algorithm to Methods.

In figure 3, c) and d) should have the same scale on the y axis.

**[Response]:** Thanks for the suggestion. We have corrected all figures.

In the Sweden analysis of Figure 6, in my opinion it is somewhat disingenuous to directly input the raw data into EpiEstim with small window sizes, as in reality, a reasonable researcher would always try to smooth out the zeros and obvious reporting errors in this dataset before entering into EpiEstim (of course, the difference is that this smoothing would be by hand and thus require more of an arbitrary choice, whereas the described method does this without arbitrary choices)

**[Response]:** Thanks for the suggestion. We have removed this improper comparison.
