# Supplementary material for: Bayesian data assimilation for estimating instantaneous reproduction numbers during epidemics: Applications to COVID-19
Source: PLoS Comput Biol. 2022 Feb 23;18(2):e1009807. doi: 10.1371/journal.pcbi.1009807 (PMC8923496; doi:10.1371/journal.pcbi.1009807)
Supplement: Attachment — Submitted filename: response_to_reviewers.docx [file pcbi.1009807.s005.docx]

Dear editor

Here, we provide our responses to the review comments and descriptions of the changes we have made in the manuscript.

**Reviewer #1:**

Major comments

1) Inconsistency between manuscript title and overall content

The new title makes the scope of the study clearer.

The modifications in the introduction have preserved the 3 points of interest to the authors (lagging, averaging, uncertainty) and reads nicely, albeit for some typos (L155 “impacts”, L200 “the rapidly changing”, L209 “arrival” -> “availability”, L215 “estimates”, L218 “comes”). It is now clearer in the manuscript that the object of this work is to tackle the rapid spread of an emerging infectious disease, in a context of rapidly evolving control measures by policymakers. In this respect, the changes address my initial comment and I am satisfied by these.

[Reponses]: Typos are fixed.

2) Proper definition of Rt

The authors have addressed my concern about correctly defining Rt throughout the

manuscript

3) Absence of internal validation and single choice for external validation

[Comment]: Could the author explain why Supplementary Table 2 doesn’t have estimates

for Jt with EpiEstim? If these can be calculated, they should be included.

[Reponses]: Unlike EpiNow2 and our method, EpiEstim does not estimate Jt. Thus, we didn’t include it in Table2.

4) Possible over-interpretation of model fits

[Comment #1]: The Python notebook in the GitHub repository has not been updated to reflect the changes in how Jt is generated (Code block In [5]). This should be done to avoid misleading users who would not be auditing the code and wrongly assume that EpiEstim is vastly outperformed when it actually isn’t.

[Reponses]: We have updated codes to the GitHub repository

[Comment #2]: In their revision, the authors have modified the x-axis scales in Fig 3 (in accordance with more data being now available in worldwide COVID time-series? Or is that a pure artificial attempt at showing multiple scenarios, with a first outbreak followed by two abrupt decreases in transmission (e.g. curfew then lockdown) and an abrupt increase weeks later e.g. abandon of control measures?).

The panels are however not all consistent with each other, one showing curves for DARt, EpiEstim and EpiNow2 (3B), while some others only show DARt and EpiNow (3D, 3E). I believe all three benchmarked methods should present equally their performance. Is there a particular reason the authors decided to drop EpiEstim curves from panels 3D and 3E? These were available pre-revision respectively in panels 3B and 3C.

[Reponses]: The reason of modifying the x-axis scales in Figure 3 from simulation experiments is that we want to show multiple scenarios.

For Figure 3D and 3E, we didn’t include EpiEstim results since EpiEstim dose not estimate Jt. Instead, it uses raw or shifted Ct for estimation. In our pre-revision figure, we use the shifted Ct to represent Jt for EpiEstim. In Figure 3D&E, since we focus on showing estimation results, we don’t include shifted Ct.

[Comment #3]: In their revised manuscript, the authors state that:

- L493–494: (paraphrased) “piNow Jt does not align nicely with the simulated curve”. This is a bit of an overstatement: rather, EpiNow Jt demonstrates that it is somewhat “over-smoothed”. In particular, I would be interested to see the cumulated curves corresponding to these time-series: I am reasonably sure that the total cumulative cases would be near identical, with the difference in peak “width” (wider for EpiNow) possibly compensating for its underestimation of point-estimate values

- L496–498: this time, the authors rightly point that DARt outperforms EpiNow2. It should still be noted that EpiNow2 point-estimates seem to fall within DARt 95%-CrI.

Overall, I believe the message that DARt outperforms other methods should still be toned down, in favor of its clear ability to detect abrupt changes in transmission.

[Reponses]: Thanks for the comments. For Jt estimation from EpiNow2, we replace the statement about misalignment with statements about over-smoothed.

[Comment #1]: Comparing the two versions of Fig 4 (before/after revision), I note that the x-axis have been adjusted for all 4 locations (likely resulting for more data being available worldwide):

- Wuhan: 16/12 to 02/03 became 20/12 to 26/02. The change here is hardly noticeable in terms of data.

- Hong Kong: 03/07 to 04/12 became 14/11 to 13/03. The dropped period seemed to consist in a high transmission period. Why was that period discarded?

- UK: 09/03 to 05/04 became 26/12 to 21/08 hence now including the 1st wave. It feels sensible to add more data to benchmark DARt rather than remove some as is the case for Hong Kong

- Sweden: 07/03 to 12/12 became 06/01 to 01/09. I can’t entirely make sense of this change.

I would appreciate if the authors could clarify why these data periods have been changed between initial submission and the first revision, as well as confirm the validity of the x-axis scale for each panel (esp. Sweden, the scales before and after revision don’t seem to match).

[Reponses]: We are afraid that this confusion might be caused by the fact that we did not include the year information in Figure 4 (Wuhan: 20/12/2019 to 26/02/2020, Hong Kong: 14/11/2020 to 13/03/2021; UK: 26/12/2020 to 21/08/2021; Sweden: 06/01/2021 to 01/09/2021.) We have updated the results with the latest data. Specifically, Hong Kong’s recent wave is during the period from 14/11/2020 to 13/03/2021; and there are no significant cases afterwards. For Sweden, the original results were from 07/03/2020 to 12/12/2020; we have now updated with the data collected in 2021. Figure 4 is updated with the year information included.

**Reviewer #2:**

Major comment:

1. The comparison between DARt, EpiNow, and EpiEstim is no longer "apples to apples," as both DARt and EpiNow are implemented taking into account an observation delay, but EpiEstim is now implemented as "plug and play" with no consideration of observation delay. In the first submission, EpiEstim was used in conjunction with Richardson-Lucy deconvolution to take into account observation delays in computing the incident infections before computing Rt. If the authors want to separate the issue of inferring incident infections using deconvolution from the accuracy of the computation of Rt itself in their application of EpiEstim, they could still take into account observation delays to align time values properly using a more simplistic method such as subtracting the mean or median of the observation delay distribution from the reported times. Some sort of correction for the observation delay still needs to be made for the EpiEstim Rt calculations as otherwise issues with inferring the shape of Rt are intermixed with issues with inferring the timing in Rt in EpiEstim but not in the other two comparisons, making conclusions about the comparison with EpiEstim misleading.

[Reponses]: Thanks for the comments. We have shifted curves from EpiEstim with the median delay time.

Minor comments:

2. The main text's writing needs to be significantly edited. Although the authors definitely made an effort to address writing issues since the first submission, this submission still has many grammatical and language issues throughout. The text of the Supplement is written much more clearly.

[Reponses]: We have made some further improvements.

3. The word "easement" needs to be replaced in the main text and supplement (say, with "easing" of restrictions) because its meaning is currently misused.

[Reponses]: Thanks for the advice. We have revised them.

4. Overall, moving the details of the algorithm from the "Results" to the "Methods" as suggested in the first round of reviews has improved the flow and readability; however, now some aspects of the "Discussion" don't make sense, as they aren't explained in the text until after the Discussion section. Specifically:
- particle filtering needs to be mentioned in the algorithm summary in "Results" in order for it to be mentioned in the "Discussion and Conclusion" section
- same for the definition of M_t

[Reponses]: Thanks for the comments. We have updated the beginning of the “Results” section.

5. Replace "the Bayes rule" with "Bayes' rule" throughout

[Reponses]: done.

6. I still feel that the discussion of reporting issues with the Sweden data has issues (lines 364-371). The periodicity and predictability of the under-reporting and subsequent correction are not the same as random noise, so I don't think it is appropriate to say that the Sweden comparison shows robustness to "observation noise".

[Reponses]: We have replaced "observation noise" with “fluctuations in observations”.

7. The formatting of citations in the text is awkward. For example, when multiple citations are listed[A], [B-E], the comma outside the bracket is unclear. One way of fixing this would be to instead write[A, B-E], all in brackets. Moreover, although in the text usually there is no space between the text and the citation[example], sometimes there is a space [example 2]. Please check this for consistency throughout.

[Reponses]: Thanks for the comments. We have updated them.

8. Throughout the text and supplement the word "synthesised" is used where the word "synthetic" would be more appropriate/conventional.

[Reponses]: We have changed this word.

9. It's interesting that the EpiNow2 results are consistently over-smoothed. Is this because the analysis uses default parameters or is it impossible to improve this even by choosing parameters more deliberately? Please at least comment on this in a sentence when discussing EpiNow results (preferably, include analysis in a supplemental figure).

[Reponses]: We have tried different modes of EpiNow2 including: 1) the default mode using a non-stationary Gaussian process to estimate the time-varying reproduction number and then infer infections, 2) a stationary Gaussian process model which is faster to estimate but currently gives reduced performance for real time estimates, and 3) the model inferring infections using back-calculation and then calculating the time-varying reproduction number. All the modes show over-smoothed results. Thus, we show the results from the default settings in the manuscript. We have added this statement in the supplementary document.

10. Dates need to be consistently formatted - for example in line 317, "21st of Jan. 2020" is different from later date formatting. Saying "Jan. 21, 2020" or "1/21/2020" would be more in line with common usage.

[Reponses]: We have reformatted.

11. Consider providing the Supplement as a .pdf: Since the Supplement is provided in a .docx instead of .pdf as it stands, much of the formatting is lost when I open it on my machine.

[Reponses]: We will submit the pdf version.

12. In Supplementary Table 2, Rt-mean, Rt-sd, Jt-mean and Jt-sd are not the best notation because these represent *differences* between the synthetic and estimated values. I would suggest using notation that indicates this, such as $\Delta$Rt-mean, $\Delta$Rt-sd, etc.

[Reponses]: Thanks for the advice. We have changed the notation in Supplementary Table 2.

13. In Supplementary Table 2, I expect that the EpiEstim row will change if an effort is made to take into account observation delays, as suggested in the major comment.

 [Reponses]: We have updated this table.

**Additional Editorial comments:**

We suggest the authors develop a title that better reflects the focus of the study. Otherwise, interested readers may not be able to find this paper. We advise against using the word "evolution" in the title, since in biology that generally means the Darwinian process of mutation and selection, which is not modeled here. Since you have specifically focused on a method for estimating Rt during emerging epidemics, we would recommend replacing this word with "growth" or "real-time reproduction number" or something similar. For example, "Bayesian data assimilation for estimating effective reproduction numbers during epidemics: applications to COVID-19". Similar comments apply to the wording used in the abstract. In the abstract, symbols should not be used before they are defined. Please use words instead of symbol for "Rt" when it's first introduced

 [Reponses]: Thanks for your advice on changing the title to be more informative. The new title is “Bayesian data assimilation for estimating instantaneous reproduction numbers during epidemics: applications to COVID-19”. Replace the word “evolution” in the abstract.
